# P-type Na^+^/K^+^ ATPases essential and nonessential for cellular homeostasis and insect pathogenicity of *Beauveria bassiana*

**DOI:** 10.1080/21505594.2020.1836903

**Published:** 2020-10-24

**Authors:** Ya-Ni Mou, Ben-Jie Gao, Kang Ren, Sen-Miao Tong, Sheng-Hua Ying, Ming-Guang Feng

**Affiliations:** a MOE Laboratory of Biosystems Homeostasis & Protection, Institute of Microbiology, College of Life Sciences, Zhejiang University, Hangzhou, Zhejiang, China; b College of Agricultural and Food Science, Zhejiang A&F University, Lin’an, Zhejiang, China

**Keywords:** entomopathogenic fungi, Na^+^/K^+^ pump genes, cellular cation homeostasis, vacuolar acidification, culture acidification, stress response, virulence

## Abstract

ENA1 and ENA2 are P-type IID/ENA Na^+^/K^+^-ATPases required for cellular homeostasis in yeasts but remain poorly understood in filamentous fungal insect pathogens. Here, we characterized seven genes encoding five ENA1/2 homologues (ENA1a–c and ENA2a/b) and two P-type IIC/NK Na^+^/K^+^-ATPases (NK1/2) in *Beauveria bassiana*, an insect-pathogenic fungus serving as a main source of fungal insecticides worldwide. Most of these genes were highly responsive to alkaline pH and Na^+^/K^+^ cues at transcription level. Cellular Na^+^, K^+^ and H^+^ homeostasis was disturbed only in the absence of *ena1a* or *ena2b*. The disturbed homeostasis featured acceleration of vacuolar acidification, elevation of cytosolic Na^+^/K^+^ level at pH 5.0 to 9.0, and stabilization of extracellular H^+^ level to initial pH 7.5 during a 5-day period of submerged incubation. Despite little defect in hyphal growth and asexual development, the Δ*ena1a* and Δ*ena2b* mutants were less tolerant to metal cations (Na^+^, K^+^, Li^+^, Zn^2+^, Mn^2+^ and Fe^3+^), cell wall perturbation, oxidation, non-cation hyperosmolarity and UVB irradiation, severely compromised in insect pathogenicity via normal cuticle infection, and attenuated in virulence via hemocoel injection. The deletion mutants of five other ENA and NK genes showed little change in vacuolar pH and all examined phenotypes. Therefore, only ENA1a and ENA2b evidently involved in both transmembrane and vacuolar activities are essential for cellular cation homeostasis, insect pathogenicity and multiple stress tolerance in *B. bassiana*. These findings provide a novel insight into ENA1a- and ENA2b-dependent vacuolar pH stability, cation-homeostatic process and fungal fitness to host insect and environment.

## Introduction

Filamentous fungal insect pathogens, particularly *Beauveria bassiana*, serve as main sources of fungal insecticides and acaricides [1]. The active ingredients of such pesticides are usually formulated conidia that are sensitive to environmental stresses, such as high temperature, solar UV irradiation, ambient pH and metal cations in host habitats [2–4]. Thus, their field efficacy and stability rely largely upon resistance/tolerance of formulated cells to outdoor adversity, making it necessary to fully understand molecular mechanisms of fungal responses to stress cues of different types. While arrays of stress-responsive signaling and effector genes in various pathways are functionally important for fungal adaptation to insect hosts and their diverse habitats [5–8], Na^+^/K^+^ pumps remains poorly understood in fungal insect pathogens.

Fungal cation pumps constitute a large super-family of plasma membrane (P-type) ATPases, classified to five families (Types I–V), which are critical for cellular homeostatic processes and responses to cation and pH changes [9], and considered to take part in pathophysiological processes and act as targets of antifungal drugs [10]. Among those, P-Type II ATPases have specificity to Ca^2+^, K^+^ and Na^+^ and fall into five subfamilies, namely Type IIA or SERCA (sarcoplasmic-endoplasmic reticulum Ca^2+^ ATPases), Type IIB or PMCA (plasma membrane Ca^2+^ ATPases), Type IIC or NK/HK (Na^+^/K^+^/H^+^ antiporters), Type IID or ENA (Na^+^/K^+^ ATPases) and Type IIE or ACU ATPases [11,12]. In *Saccharomyces cerevisiae*, two sodium pumps found in an early study were named ENA1 and ENA2 in terms of the Latin *exitus natru* (exit of sodium) and proven to mediate cell tolerance to Na^+^, Li^+^ and alkaline pH [13]. Subsequent studies revealed an existence of ENA or ENA-like ATPases in yeast and filamentous fungi [14], including NHA1 as an alkaline cation antiporter to mediate an efflux of Na^+^, K^+^ and/or H^+^ in *S. cerevisiae* [15] and ACU1/2 as novel K^+^/Na^+^ transporters phylogenetically distant from ENA in *Ustilago maydis* [16]. Homologous ENA1/2 genes were reported to have similar roles in mediating cell responses to K^+^/Na^+^ and pH changes in various yeasts, such as *Saccharomyces occidentalis* [14], *Zygosaccharomyces rouxii* [17] and *Debaryomyces hansenii* [18], and serve as essential regulators of alkali cation homeostasis in *Hortaea werneckii* [19] and of cell tolerance to Na^+^, K^+^ and high pH in *U. maydis* [20]. In *Cryptococcus neoformans*, deletion of ENA1 gene resulted in a complete loss of virulence to mice and increased cell sensitivity to alkaline pH but null responses to stress cues of concentrated Na^+^, K^+^, Li^+^ and sorbitol [21]. In *Candida glabrata*, ENA1 and CNH1 ATPases were evidently involved in Na^+^ detoxification and K^+^ homeostasis, respectively, and the latter showed lower Na^+^/H^+^ antiporter activity than the homologue NHA1 in *S. cerevisiae* despite a similarity in broad substrate specificity [22].

Compared to intensive studies in yeast or yeast-like fungi, ENA and ENA-like ATPase genes have been rarely explored in filamentous fungi until recently. In *Fusarium oxysporum*, transcriptional activation of an ENA gene at high Na^+^ levels and alkaline pH was shown to be regulated by PacC [23], a transcription factor that functions in the pH-responsive Pal/Rim pathway essential for fungal response to ambient pH [24,25]. Another ENA gene (*MaENA1*) has proved functional in cell tolerance to Na^+^, heat and UVB irradiation and transcriptional activation of many stress-responsive genes in the locust-pathogenic fungus *Metarhizium acridum* [26]. Two ENA genes in the plant endophyte *Serendipita indica* are involved in an efflux of Na^+^/K^+^ and hence an increase of plant tolerance to Na^+^ under saline conditions [27]. These studies demonstrate not only conserved roles of ENA genes in maintenance of intracellular cation homeostasis in yeast and filamentous fungi but also a linkage of their functions with fungal survival in hosts and host habitats. However, the previous studies paid little attention to P-Type IIC or NK/HK ATPase genes and a linkage of an ENA-mediated Na^+^/K^+^ efflux with pH change in the vacuoles where an array of cellular events take place, such as alkali cation storage, vacuolar protein sorting, cellular homeostasis, signaling, and stress responses crucial for fungal adaptation to host and environment [28].

We found up to five Type IID/ENA ATPases homologous to the yeast ENA1/2 and also two Type IIC/NK ATPases in the genomes of *B. bassiana* [29] and other entomopathogenic fungi [30–32]. It is unknown whether so diverse ENA genes are involved in cellular Na^+^/K^+^/H^+^ homeostatic processes of fungal insect pathogens and their adaptation to hosts and host habitats. Of those, only *MaENA1* in *M. acridum* has been functionally explored [26]. In *B. bassiana*, P-Type IIA and IIB Ca^2+^ ATPase genes and five vacuolar Ca^2+^ exchanger genes have been shown to play important, but differential, roles in intracellular Ca^2+^ homeostasis, multiple stress tolerances, and phenotypes associated with the fungal potential against insect pest [33–35]. The Na^+^/H^+^ antiporter Nhx1 has also proved essential for vacuolar homeostasis, conidiation, host infection via cuticular penetration, and virulence-related cellular events post-infection [36]. The previous studies suggest a link between cellular cation homeostasis and vacuolar events critical for the asexual life of *B. bassiana*. We hypothesize that some, if not all, of those ENA and NK genes are likely involved in the important linkage. This study sought to elucidate functions of seven genes encoding three ENA1 (ENA1a–c), two ENA2 (ENA2a/b) and two NK (NK1/2) homologues in *B. bassiana* through multiple analyses of single-gene deleted and complemented mutants.

## Results

### Sequence features of ENA and NK ATPases in *B. bassiana*

Five ENA homologues were located in the *B. bassiana* genome [29] through BLASTp analysis with the queries of the yeast ENA1 and ENA2 sequences. They are phylogenetically close, and hence homologous, to ENA1 and ENA2 in examined yeasts and filamentous fungi. Thus, three ENA1 and two ENA2 homologues are named as ENA1a–c (EJP65015, EJP62002 and EJP68283) and ENA2a/b (EJP65986 and EJP63485) (Supplementary Fig. S1). Two other ENA-like proteins found in the fungal genome fall into the same clade of NK1/PAT1 and NK2/PAT2 in other fungal genomes and are named as NK1 (EJP64376) and NK2 (EJP66197). The *B. bassiana* ENA and NK homologues also exist in other fungal insect pathogens (Supplementary Fig. S2), hinting at greater ENA diversity in entomopathogenic fungi than in non-entomopathogenic fungi. The ENA and NK homologues are more or less distinct in molecular feature from one to another, and all of them are predicted to localize to the plasma membrane due to 10 or 11 transmembrane regions distributed throughout their sequences (Supplementary Table S1).

**Figure 1.**
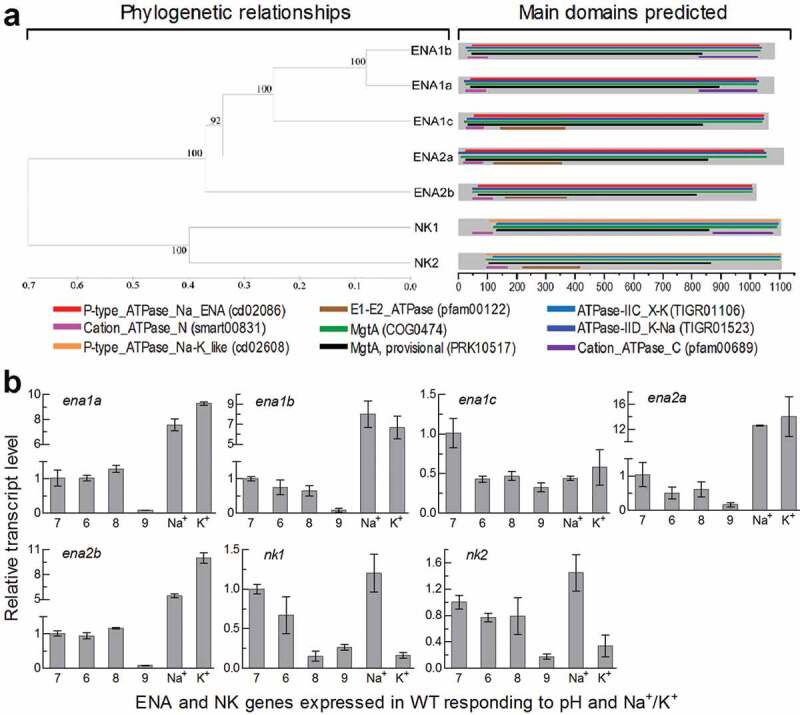
Recognition and transcriptional profiles of ENA and NK ATPases in *B. bassiana*. (a) Phylogenetic relationships of ENA1a-c, ENA2a/b and NK1/2 revealed by cluster analysis and their functional domains predicted at https://www.ncbi.nlm.nih.gov/Structure/. The bootstrap values of 1000 replications are given at nodes. Scale: branch length proportional to genetic distance assessed with a neighbor-joining method in MEGA7 at http://www.megasoftware. net/. (b) Relative transcript levels of five ENA and two NK genes in the 3-day-old cultures of a wild-type strain (WT) grown at 25°C in CDB, which was adjusted to pH 6.0, 7.0, 8.0 and 9.0 respectively or supplemented with 0.7 M of NaCl or KCl and adjusted to pH 7.0, and calculated with respect to the standard at pH 7.0. Error bars: SD of the mean from three cDNA samples assessed by qPCR analysis with paired primers (Table S2)

In *B. bassiana*, five ENA homologues are similar in structure and share multiple functional regions, including P-type_ATPase_Na_ENA (fungal-type Na^+^-ATPase), ATPase-IID_K-Na (K^+^/Na^+^ efflux P-type ATPase), MgtA (Mn^2+^-transporting ATPase) and Cation_ ATPase_N (cation transporter/ATPase) (Figure 1(a)). Among those, ENA1a and ENA1b also share Cation_ATPase_C (C-terminal cation transporting ATPase), contrasting to an N-terminal E1-E2_ATPase present in ENA2a/b and ENA1c. However, the sequence of ENA1c is more similar to those of ENA1a/b than of ENA2a/b (Table S1). NK1 and NK2 feature the functional regions ATPase-IIC_X-K (Na^+^/H^+^ efflux or K^+^ uptake antiporter) and P-type_ATPase_Na-K_like (α-subunit of Na^+^/K^+^-ATPase) and hence distant in phylogeny from the ENA ATPases.

### Transcriptional profiles of ENA and NK genes in *B. bassiana*

The ENA and NK genes were differentially expressed in the 3-day-old cultures of the wild-type strain *B. bassiana* ARSEF 2860 (designated WT) grown at optimal 25°C in Czapek-Dox broth (CDB), which was adjusted to initial pH 6.0–9.0 or supplemented with 0.7 M of NaCl or KCl and then adjusted to initial pH 7.0. Compared to a standard at pH 7.0, transcript levels of four ENA genes not including *ena1c* were sharply repressed at alkali pH 9.0, largely upregulated in the presence of Na^+^ or K^+^ but insignificantly or less affected at pH 6.0 or 8.0 (Figure 1(b)). Expression of *ena1c* was differentially suppressed under acidic, alkaline and Na^+^/K^+^-stressed conditions. Two NK genes were largely suppressed at pH 9.0 and 0.7 M K^+^ but slightly upregulated at 0.7 M Na^+^. These data demonstrated that all ENA and NK genes were much more sensitive to the alkali pH than a pH range closer to the neutral level. The Na^+^- and K^+^-upregulated ENA genes at pH 7.0 could be more likely involved in an efflux of alkali cations critical for cellular homeostasis under normal conditions.

### ENA and NK genes essential and nonessential for cellular homeostasis

Each ENA or NK gene was deleted from WT by homologous recombination of its 5′ and 3′ coding/flanking fragments separated by the *bar* marker and rescued in an identified deletion mutant by ectopic integration of a cassette comprising its full-length coding/flanking sequence and the *sur* marker, as described in Methods. The gene-deleted and complemented strains were identified through PCR and Southern blot analyses (Supplementary Fig. S3) with paired primers and amplified probes (Supplementary Table S2) and then evaluated for their changes in cellular H^+^, Na^+^ and K^+^ levels with respect to the parental WT strain.

**Figure 2.**
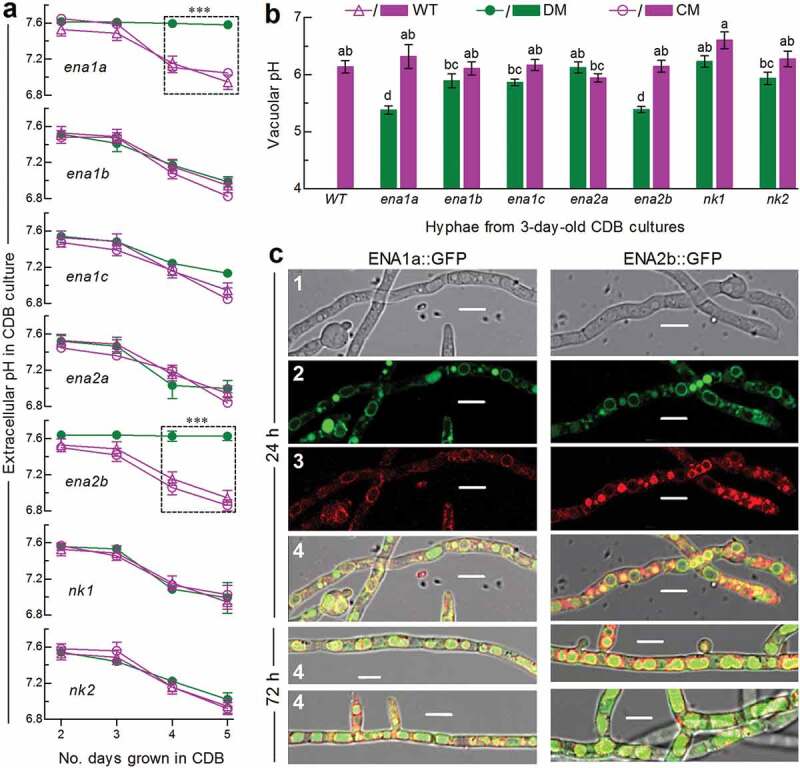
Roles of ENA and NK genes in homeostatic processes of cellular H^+^ in *B. bassiana*. (a) Extracellular pH (H^+^) levels quantified from the submerged cultures of single-gene deletion mutants (DM), complemented mutants (CM) and WT during a 5-day incubation at 25°C in CDB at initial pH 7.5 (unadjusted). ***P < 0.001 between DM and WT or CM. (b) Vacuolar pH (H^+^) levels quantified from fluorescent dye-stained hyphae at the excitation/emission wavelengths of 450/535 and 490/535 nm under a confocal microscope. The examined hyphae were collected from 3-day-old CDB cultures. Means marked with different lowercase letters differ significantly (Tukey’s HSD, *P* < 0.05). Error bars: SD of the mean from three replicates of independent cultures (**a**) or of 15 vacuoles (**b**). (c) LSCM images (scale bars: 10 μm) for subcellular localization of GFP-tagged ENA1a and ENA2b fusion proteins (shown in green) in the WT hyphae collected from 24-h- and 72-h-old SDBY cultures and stained with FM4-64 (shown in red). Panels 1–4 are bright, expressed, stained and merged views of the same microscopic field. Note that both ENA1a::GFP and ENA2b::GFP molecules accumulate heavily in the vacuoles

As an indicator of extracellular H^+^ level, pH was measured daily from the submerged cultures of a 10^6^ conidia/ml CDB at an unadjusted pH of ~7.5 during a 5-day period of shaking incubation at 25°C. The pH values measured from the cultures of Δ*ena1a* and Δ*ena2b* were stabilized persistently to the initial level and significantly higher (Tukey’s HSD, *P* < 0.05) than those in the 4- and 5-day-old cultures of their control (complemented and WT) strains, contrasting to similar pH-declining trends over the time of incubation among other deletion mutants and control strains (Figure 2(a)). Obviously, *ena1a* and *enA2b* were involved in the mediation of extracellular H^+^ level and culture acidification. To explore a linkage between extracellular and intracellular H^+^ levels, a calibrated equation of linear correlation between vacuolar pH and green versus red fluorescence intensity ratio at excitation/emission wavelengths of 450/535 and 490/535 nm (Supplementary Fig. S4) was established as described previously [36]. The equation was used to quantify vacuolar pH from the hyphae stained with pH-sensitive and membrane-specific dyes. The used hyphae were collected from the 3-day-old CDB cultures, in which similar pH levels were above 7.3 for all tested strains at the sampling time. As a result, vacuolar pH was lowered to 5.38 (± 0.06) in both Δ*ena1a* and Δ*ena2b*, contrasting to a similar level of 6.14 ± 0.20 in the rest mutants and control strains (Figure 2(b)). For the two mentioned mutants, acidified vacuolar lumen was followed by a subsequent blockage of culture acidification, implicating a disturbance of intracellular cation homeostasis.

**Figure 3.**
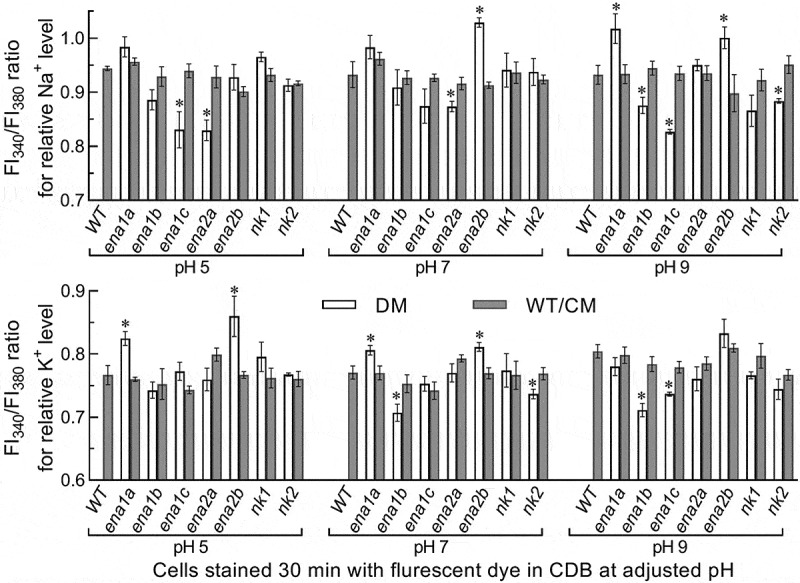
Roles of ENA and NK genes in homeostatic processes of cellular Na^+^ and K^+^ in *B. bassiana*. Cytosolic Na^+^ (upper panel) and K^+^ (lower panel) levels are presented as ratios of fluorescence intensities (FI), which were quantified at the alternating excitation wavelengths of 340/380 nm from the cells incubated at 25°C for 30 min in CDB at indicated pH levels and stained with 10 μM of Na^+^- and K^+^-specific fluorescent probes (SBFI-AM and PBFI-AM) respectively. The asterisked means of deletion mutants differ significantly from those of corresponding control strains not marked (Tukey’s HSD, *P* < 0.05). Error bars: SD of the mean from three replicates of independent cultures

The above speculation was examined in two experiments. First, the WT hyphae expressing green fluorescence-tagged ENA1a and ENA2b fusion proteins, respectively, were stained with the membrane-specific dye FM4-64 and analyzed with laser scanning confocal microscopy (LSCM). As a result, both fusion proteins appeared on the membranes of organelles including vacuoles at the time of 24-h incubation in SDBY and accumulated heavily in the vacuoles of the hyphae collected from 72-h-old cultures (Figure 2(c)). The vacuolar localization of either fusion protein was well defined by the vacuolar membranes shown as an orange color of expressed green overlapped with the dye color (shown in red). These observations revealed both transmembrane and vacuolar activities of either ENA1a or ENA2b and an involvement of each in vacuolar events. Second, intracellular or cytosolic Na^+^ and K^+^ levels were quantified from the 24-h-old cultures of a 10^7^ conidia/ml CDB adjusted to initial pH 5.0, 7.0 and 9.0 using Na^+^- and K^+^-specific florescence probes, respectively. As denoted by the ratios of fluorescence intensities at the excitation/emission wavelengths of 340/380 nm (Figure 3), cytosolic Na^+^ level increased significantly in Δ*ena1a* and Δ*ena2b* at alkali pH 9.0 but only in Δ*ena2b* at pH 7.0. Cytosolic K^+^ levels of the two mutants also increased significantly under acidic and neutral conditions but were not affected at pH 9.0. However, neither Na^+^ nor K^+^ level was elevated in the cells of other deletion mutants. Instead, the Na^+^ level was lowered in Δ*ena1b* and Δ*nk2* at pH 9.0, in Δ*ena1c* at pH 5.0 or 9.0, and in Δ*ena2a* at pH 5.0 or 7.0. In addition, a decreased K^+^ level was observed in Δ*ena1b* and Δ*nk2* at pH 7.0 and in Δ*ena1b* and Δ*ena1c* at pH 9.0.

Taken together with the transmembrane and vacuolar activities of ENA1a and ENA2b, pH-responsive changes of cytosolic Na^+^/K^+^ levels in Δ*ena1a* and Δ*ena2b* implicate that an efflux of Na^+^ and/or K^+^ from the vacuoles harboring such alkali cations resulted in their vacuolar acidification and an increased afflux into the cytosol. Also, an increased efflux of Na^+^/K^+^ accumulated in the cytosol led to stabilization of pH or extracellular H^+^ levels in the cultures of both mutants via neutralization of organic acids (extracellular H^+^) produced by the cultures [25]. Thus, only *ena1a* and *ena2b* are essential for homeostatic processes of cellular Na^+^, K^+^ and H^+^ in *B. bassiana*. Three other ENA and two NK genes seem to be redundant for the homeostatic processes. This is in evidence with their deletion mutants, which exhibited negligible changes in vacuolar pH, rare elevation in cytosolic Na^+^/K^+^ level, and the same culture pH-declining trends over the time of submerged incubation as seen in the control strains.

### Roles of ENA and NK genes in growth, conidiation and multiple stress responses

Hyphal growth and development for aerial conidiation are crucial for the lifecycle *in vitro* and *in vivo* of an asexual insect mycopathogen like *B. bassiana*. Radial growth on the plates of rich SDAY (Sabouraud dextrose agar plus yeast extract) and minimal CDA (i.e., CDB plus agar) showed no significant variability (*P* > 0.2 in analysis of variance) among all tested strains (Supplementary Fig. S5(a) and S5(b)). Conidial yields from the 9-day-old SDAY cultures initiated by spreading 100 μl aliquots of a 10^7^ conidia/ml suspension and incubated at an optimal regime were not significantly different (Tukey’s HSD, *P* > 0.05) between each deletion mutant and its two control strains (Supplementary Fig. S5(c)). Conidial germination at 25°C was delayed differentially in the deletion mutants not including Δ*nk2* in comparison to median germination time (GT_50_) estimated from the control strains (Supplementary Fig. S5(d)). The germination defects were moderate in both Δ*ena1a* and Δ*ena2b* (GT_50_ prolonged by ~3.5 h) and light in other deletion mutants (GT_50_ prolonged by ~1 h or less). Also, median lethal dose (LD_50_) for conidial resistance to UVB irradiation was lowered only in the two mentioned mutants (Supplementary Fig. S5(e)). Thus, five ENA and two NK genes are all dispensable for the fungal growth and conidiation.

**Figure 4.**
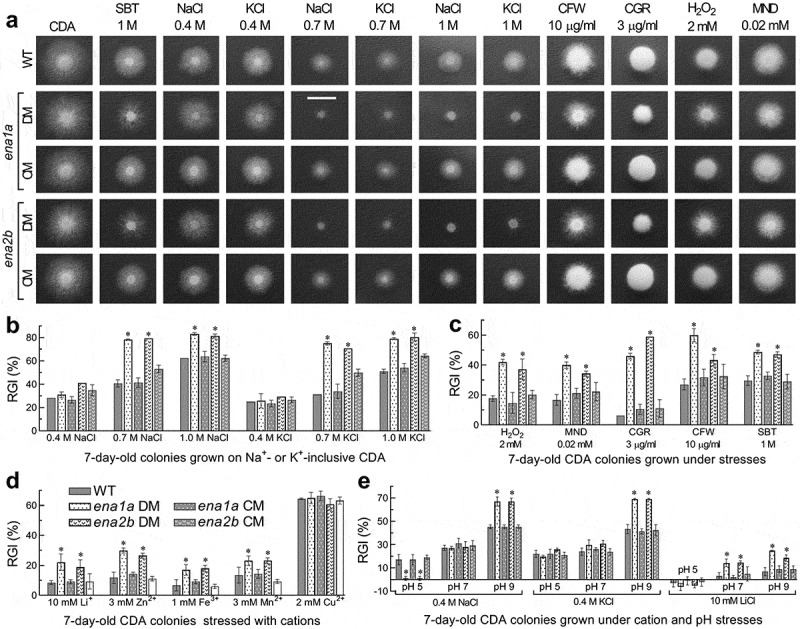
Essential roles of *ena1a* and *ena2b* in *B. bassiana* responses to multiple stresses during a 7-day growth at 25°C in a light/dark cycle of 12:12 h. (a) Images (scale bar: 10 mm) for 7-day-old colonies of single-gene deletion mutants (DM), complemented mutants (CM) and WT co-cultivated with or without chemical stressors in CDA. (b–d) Relative growth inhibition (RGI) percentages of fungal colonies on pH-unadjusted CDA plates supplemented with indicated concentrations of NaCl, KCl, H_2_O_2_, MND (menadione), CGR (Congo red), CFW (calcofluor white), SBT (sorbitol), and five metal cations respectively. (e) RGI values of fungal colonies on CDA with initial pH adjusted to 5.0, 7.0 and 9.0 after 0.4 M NaCl, 0.4 M KCl or 10 mM LiCl was added for response to a combination of each cation with pH change. Each colony was initiated by spotting 1 μl of a 10^6^conidia/ml suspension. RGI values were computed with respect to a control free of any stress cue in the pH-unadjusted CDA (**b–d**) or the CDA at pH 7.0 (**e**). Asterisked means in each bar group differ significantly from those not marked (Tukey’s HSD, *P* < 0.05). Error bars: SD of the mean from three replicates

Despite little influence on normal growth and conidiation, either *ena1a* or *ena2b* deletion resulted in increased sensitivities to stress cues during a 7-day colony growth on pH-unadjusted CDA at 25°C (Figure 4(a)). This was in contrast with equal or nearly equal stress responses of all other tested strains (Supplementary Fig. S6). Based on relative growth inhibition of fungal colony under each stress, both Δ*ena1a* and Δ*ena2b* were 19–39% more sensitive to 0.7 and 1 M of NaCl and KCl than were their control strains but showed null response to 0.4 M of either osmotic salt (Figure 4(b)). Moreover, the two mutants were compromised in tolerance to hyperosmotic stress of non-cation sorbitol (decreased by 19% and 17%), oxidative stress of menadione (23% and 18%) or H_2_O_2_ (24% and 20%), and cell wall perturbing stress of Congo red (40% and 53%) or calcofluor white (33% and 16%) (Figure 4(c)). The two mutants also were 10–18% more sensitive to Li^+^, Zn^2+^, Fe^3+^ and Mn^2+^ despite little change in sensitivity to Cu^2+^ (Figure 4(d)).

To reveal an impact of ambient pH on cellular responses to Na^+^, K^+^ and Li^+^, we assessed colony growth inhibition percentages of each strain on the CDA supplemented with 0.4 M NaCl, 0.4 M KCl or 10 mM LiCl and then adjusted to pH 5.0, 7.0 and 9.0. Compared to the control strains, both Δ*ena1a* and Δ*ena2b* became significantly more sensitive to 0.4 M of Na^+^ (~22%) and K^+^ (~26%) at alkali pH 9.0, contrasting to their null response to either cation at pH 7.0 (Figure 4(e)) as seen at unadjusted pH. Particularly, the two mutants were ~16% more tolerant to 0.4 M of Na^+^ instead of K^+^ at acidic pH 5.0 but 11–18% more sensitive to 10 mM Li^+^ under neutral and alkali conditions. However, most of the rest deletion mutants showed insignificant changes in their responses to the alkali cations at the tested pH levels (Supplementary Fig. S7). Exceptionally, Δ*ena1b* became markedly sensitive to Li^+^ at pH 9.0 and slightly sensitive to Na^+^ at pH 5.0 or K^+^ at pH 7.0. Increased tolerance to K^+^ at pH 9.0 or Li^+^ at pH 5.0 was observed in Δ*ena1c*, contrasting to Δ*ena2a* showing increased sensitivity to K^+^ at pH 7.0.

The above stress-responsive phenotypes were restored by the complementation of *ena1a* into Δ*ena1a* and of *ena2b* into Δ*ena2b*. These results indicate active roles of *ena1a* and *ena2b* in *B. bassiana* responses to not only multiple metal cations including Na^+^, K^+^ and Li^+^ but also cell wall perturbation, oxidation and non-cation hyperosmolarity, highlighting a requirement of both genes for the fungal adaptation to host and host habitat. Three other ENA and two NK genes are dispensable for the fungal responses to the mentioned stresses. The altered cell responses to low-level Na^+^/K^+^ cues under acidic and/or alkali conditions implicate that *ena1a* and *ena2b* could be involved in response to Na^+^/K^+^ in a pH-dependent fashion and also that ambient pH could be important for weak responses of other ENA genes to Na^+^ or K^+^ and sensitive response of *ena1b* to Li^+^ in *B. bassiana*.

### ENA and NK genes essential and nonessential for fungal pathogenicity and virulence

**Figure 5.**
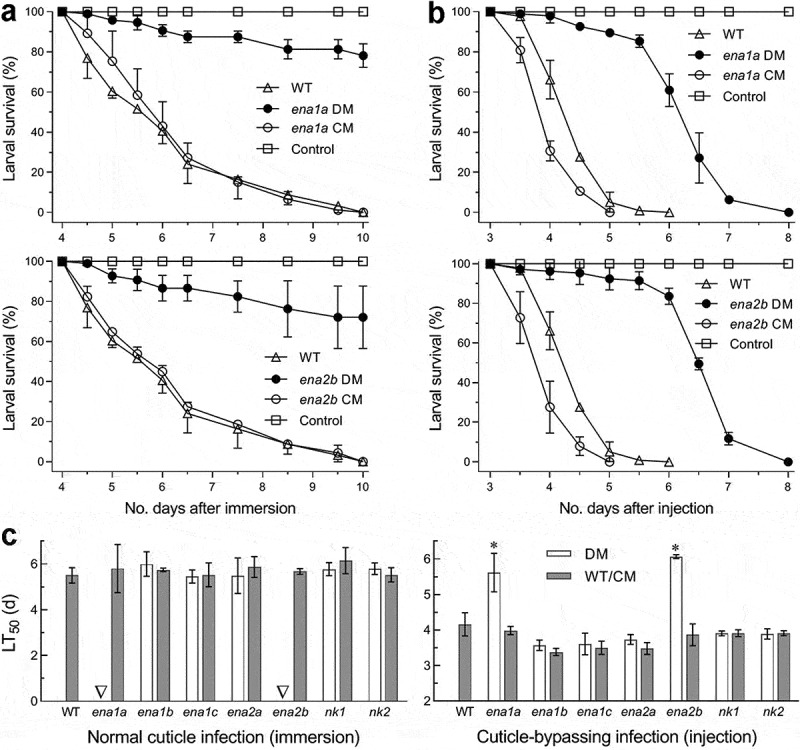
Requirements of *ena1a* and *ena2b* for pathogenicity and virulence of *B. bassiana*. (a, b) Survival trends of *G. mellonella* larvae after topical application (immersion) of a 10^7^ conidia/ml suspension for normal cuticle infection and hemocoel injection of ~500 conidia per larva for cuticle-bypassing infection respectively. (c) LT_50_ values estimated for deletion mutants (DM), complemented mutants (CM) and WT against the larvae through probit analyses of time-mortality trends derived from (a), (b) and Supplementary Fig. S8. Note that LT_50_s are unavailable for Δ*ena1a* and Δ*ena2b* (arrowed) through the normal infection, much prolonged for both mutants (asterisked, *P* < 0.001 for Tukey’s HSD test) through the cuticle-bypassing infection, but similar for all other deletion mutants and control strains in either infection mode. Error bars: SD of the mean from three replicates

Fungal insect pathogenicity is an all-or-none response of insect host to hyphal invasion and conceptually distinguished from virulence, a measurable fungal ability to cause host mycosis and death, in terms of microbial pathogenicity and virulence defined in a general sense [37]. The pathogenicity and virulence of each fungal strain against the fifth-instar larvae of greater wax moth (*Galleria mellonella*) were assayed through topical application (immersion) of a 10^7^ conidia/ml suspension for normal cuticle infection and intrahaemocoel injection of ~500 conidia per larva for cuticle-bypassing infection. As a consequence, most Δ*ena* and Δ*nk* mutants and their control strains caused 100% mortality within 10 days post-immersion or 6 days post-injection and similar time-survival or mortality trends (Supplementary Fig. S8). In contrast, the Δ*ena1a* and Δ*ena2b* mutants resulted in only 22% and 28% mortalities via the normal infection (Figure 5(a)) and much slower time-mortality trends than did the control strains via injection (Figure 5(b)). The time-mortality trends led to similar estimates of median lethal time (LT_50_) indicative of virulence or lethal action for all tested strains except the two mentioned mutants against the model insect through either infection mode (Figure 5(c)). Consequently, the LT_50_ estimates of Δ*ena1a* and Δ*ena2b* were not computable via the normal infection and increased to 5.6 and 6.1 days from a mean of 4.0 days for their control strains to cause 50% mortality via the injection, respectively. These data indicated a requirement of either *ena1a* or *ena2b* for the fungal pathogenicity through the normal route of cuticular penetration and an essential role of each in certain virulence-related cellular events after entry into host hemocoel. However, both pathogenicity and virulence of *B. bassiana* were not affected by knockout mutations of the rest ENA and NK genes.

**Figure 6.**
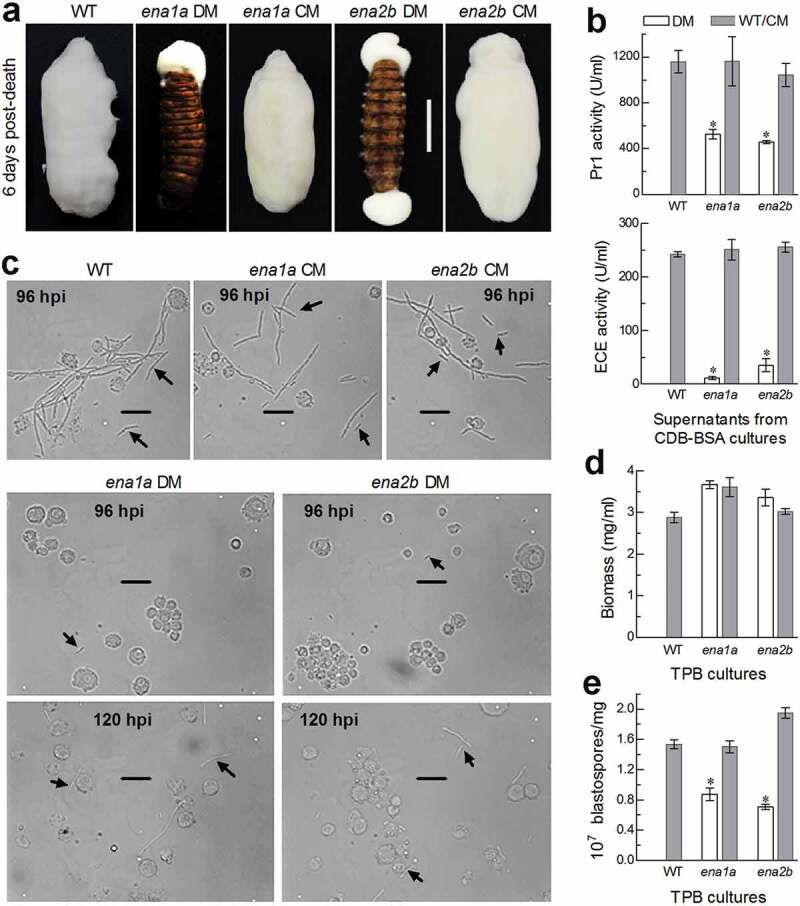
Important roles of *ena1a* and *ena2b* in cellular events critical for pathogenicity and virulence of *B. bassiana*. (a) Images (scale: 10 mm) of fungal outgrowths on *G. mellonella* larvae 6 days after death from mummification by deletion mutants (DM), complemented mutants (CM) and WT through intrahaemocoel injection. Note that two deletion mutants grew out only from insect mouth and/or anus but failed to directly penetrate through the cadaver cuticle for outgrowth. (b) Total activities of cuticle-degrading extracellular enzymes (ECE) and Pr1 proteases quantified from the supernatants of 3-day-old CDB-BSA cultures, which were initiated with 50 ml aliquots of a 10^6^ conidia/ml suspension at 25°C. (c) Microscopic images (scale: 20 μm) of hyphal bodies (arrowed) formed in the hemolymph of larvae surviving 96 or 120 h post-injection (hpi). Spherical or subspherical cells are host hemocytes. (d, e) Biomass levels and dimorphic transition rates (no. blastospores/mg biomass) measured from the 3-day-old cultures of a 10^6^ conidia/ml TPB mimicking insect hemolymph. Asterisked means are significantly different from those unmarked in each graph (Tukey’s HSD, *P* < 0.01). Error bars: SD of the mean from three replicates

Hyphal invasion into host insect requires secretion and action of extracellular (proteolytic, chitinolytic and lipolytic) enzymes including subtilisin-like Pr1 proteases to degrade and penetrate through the host cuticle [38,39]. After entry into the host hemocoel, penetrating hyphae turn into unicellular hyphal bodies (blastospores) for acceleration of fungal propagation by yeast-like budding until host mummification to death, at the time of which hyphal bodies turn back into the hyphae to penetrate again through the cuticle for outgrowth and conidiation on cadaver surfaces [40,41]. For these reasons, we explored possible roles of *ena1a* and *ena2b* in cuticular penetration critical for insect pathogenicity and post-infection dimorphic (hypha-blastospore or *vice versa*) transition enabling to accelerate host death and fungal outgrowth on cadaver surfaces. First, insect cadavers mummified by the control strains through injection were covered with a heavy layer of fungal outgrowth by the end of 6 days post-death, contrasting to an absence of fungal outgrowth on the surfaces of the cadavers mummified by Δ*ena1a* and Δ*ena2b*, which grew out only from mouth or anus (Figure 6(a)). These observations indicated that intrahaemocoel hyphae of both mutants were unable to penetrate through the cadaver cuticle for normal outgrowth, hinting at a likelihood that the two mutants could have been compromised in an ability to infect the insect through the normal route of culticular penetration. This speculation was verified with total activities of extracellular enzymes (ECEs) and Pr1 proteases quantified from the supernatants of the 3-day-old submerged cultures. As a result, total ECE and Pr1 activities were reduced by 95% and 55% in Δ*ena1a* and 86% and 61% in Δ*ena2b*, respectively, in comparison to the corresponding WT quantities (Figure 6(b)). Next, we examined hemolymph samples taken from the larvae surviving a period after injection and observed abundant hyphal bodies formed by the control strains in the samples taken 96 h post-injection (Figure 6(c)). By contrast, hyphal bodies formed by the two mutants were much less abundant even at the time of 120 h post-injection. Further, dimorphic transition rates of each strain were assessed using biomass levels and blastospore concentrations in the 3-day-old cultures grown in a trehalose-peptone broth (TPB) mimicking insect hemolymph. Despite slightly increased biomass levels (Figure 6(d)), dimorphic transition rate was reduced by 43% in Δ*ena1a* and 54% in Δ*ena2b* compared to the control strains (Figure 6(e)). Such percent reductions coincided well with 44%- and 56%-prolonged LT_50_ estimates for the two mutants to cause 50% mortality via the injection. These results indicated that both Δ*ena1a* and Δ*ena2b* were compromised in dimorphic transition and hence attenuated in virulence through the cuticle-bypassing infection.

## Discussion

ENA and NK homologues are presumable or verified Na^+^/K^+^ pumps required for cellular cation homeostasis in yeasts and non-yeast fungi [11,12]. Among five ENA and two NK genes characterized in this study, only *ena1a* and *ena2b* were essential for cellular Na^+^/K^+^/H^+^ homeostasis, environmental adaptation and insect pathogenicity of *B. bassiana*. None of them showed a substantial role in maintenance of hyphal growth and conidiation under normal culture conditions. The essential and nonessential roles of these ATPase genes in *B. bassiana* are discussed below.

Fungal vacuoles are deeply involved in many cellular events, including uptake/storage of various ions, cellular homeostasis and membrane trafficking [28], and such events rely largely upon vacuolar homeostasis mediated by vacuolar proton-translocating ATPases, namely V-ATPases [42]. In this study, essential roles of *ena1a* and *ena2b* in homeostatic processes of cellular Na^+^, K^+^ and H^+^ are tightly linked to vacuolar acidification, which occurred in the absence of either gene in *B. bassiana*. In Δ*ena1a* and Δ*ena2b*, the accelerated vacuolar acidification is inferred to result from an efflux of alkali Na^+^/K^+^ from acidifying vacuoles and hence to increase an afflux of such cations into cytosol. This inference is supported not only by both an increase of cytosolic Na^+^/K^+^ level and a marked delay of the culture acidification by an efflux of Na^+^/K^+^ more accumulated in cytosol but also by an entry of ENA1a or ENA2b into vacuoles across the vacuolar membrane due to multiple transmembrane sites predicted for either protein. First, extracellular H^+^ or pH levels in the cultures of Δ*ena1a* and Δ*ena2b* were well stabilized to initial pH 7.5 during a 5-day period of shaking incubation, contrasting to all other deletion mutants and control strains showing similar pH-declining trends over the time of incubation. In both deletion mutants, second, an increased efflux of cytosolic Na^+^/K^+^ is evidenced with increased accumulation of Na^+^ and/or K^+^ in cytosol at pH 5.0 to 9.0. Previously, knockout mutations of *pacC* and *pal* partners in the Pal/Rim pathway of *B. bassiana* resulted in delayed acidification of submerged cultures due to reduced production and/or secretion of organic acids, such as lactic acid and oxalic acid [25]. On the other hand, knockout mutation of the V-ATPase subunit H gene *vmaH* in *B. bassiana* led to acceleration of culture acidification and upregulation of several genes involved in vacuolar behavior and organic acid production [43]. Taken the present and previous results into account, we infer that the culture acidification of Δ*ena1a* or Δ*ena2b* was impeded by an afflux of N^+^/K^+^ into cytosole from the acidifying vacuoles and then an efflux of N^+^/K^+^ from cytosol to neutralize organic acids produced in the cultures. In the rest Δ*ena* and Δ*nk* mutants, vacuolar pH was maintained at the same level as seen in all control strains, and cytosolic Na^+^/K^+^ levels were rarely elevated at pH 5.0 to 9.0. Thus, it is unlikely for these deletion mutants to increase an efflux of N^+^/K^+^ from cytosol to stabilize their culture pH. Neither did they show significant changes in insect pathogenicity and multiple stress tolerances associated with the fungal adaptation to host and environment. These findings uncover a novel linkage of vacuolar H^+^ homeostasis with essential roles of *ena1a* and *ena2b* in cellular Na^+^/K^+^/H^+^ homeostasis and provide an insight into why three other ENA and two NK genes are functionally redundant in *B. bassiana*.

Cellular responses to stress cues reflect a capability of fungal adaptation to environment and are mediated by various signaling pathways in fungal insect pathogens [5–8]. In this study, only *ena1a* and *ena2b* essential for cellular homeostasis showed positive roles in maintenance of cell tolerance to metal cations, cell wall perturbation, oxidation, non-cation hyperosmolarity and UVB irradiation, and also cell responses to Na^+^, K^+^ and Li^+^ in a pH-dependent fashion. So diversified stress responses are much beyond ENA1- and/or ENA2-mediated responses to alkali cations in yeasts [14,17–19] and *U. maydis* [20], and also distinguished from null responses of *C. neoformans* ENA1 to Na^+^, K^+^, Li^+^ and sorbitol cues [20]. The diversified roles implicate that both *ena1a* and *ena2b* are crucial for the adaptation of *B. bassiana* to its broad host spectrum and diverse host habitats and also for field persistency and efficacy of the fungal formulations against arthropod pests. Previously, loss-of-function mutation of MaENA1 (EFY86649) in *M. acridum* decreased cell tolerance to Na^+^, heat and UVB irradiation and caused differential expressions of 281 genes [26]. Despite limited phenotypes disclosed, the previous study revealed transcriptional repression of many genes in the absence of *MaENA1*, including dozens involved in the activities of antioxidation, ion-binding, DNA repair and detoxification and key signaling genes acting in stress-responsive Ras-cAMP PKA, Pal/Rim, Ca^2+^/calmodulin and mitogen-activated protein kinase pathways. However, homologous *ena1a* is absent in *M. acridum* and the characterized *MaENA1* is close to *ena1b* not functional in *B. bassiana*. This implicates that *MaENA1* in *M. acridum* could function as does *ena1a* in *B. bassiana* and that functions of ENA genes could have differentiated among the lineages of fungal insect pathogens with different host spectra. If *MaENA1* was functionally close to *ena1a*, those repressed genes in the previous study [26] could help to understand diversified stress-responsive roles of both *ena1a* and *ena2b* in *B. bassiana*. Nonetheless, we speculate that the *ena1a*- and *ena2b*-mediated cellular responses to the stress cues of different types also could be associated with their roles in vacuolar homeostasis since vacuolar acidification may exert fundamental impact on a large array of cellular events [28,42]. For instance, markedly increased sensitivities of our Δ*ena1a* and Δ*ena2b* mutants to cell wall perturbing and non-cation osmotic stresses could have resulted from cell wall permeability compromised by an increased efflux of alkali Na^+^/K^+^ more accumulated in cytosol. Increased sensitivities to metal cations also concurred with vacuolar acidification when the Na^+^/H^+^ antiporter gene *nhx1* lost function [36].

Pathogenicity and virulence represent a capability of fungal adapatation to host and were rarely associated with functions of ENA genes in previous studies. Exceptionally, deletion of *ena1* in *C. neoformans* led to abolished pathogenicity to mice as well as increased cell sensitivity to alkaline pH [21]. In *B. bassiana*, essential roles of both *ena1a* and *ena2b* in normal host infection and insect pathogenicity are well indicated by an uncomputable LT_50_ for their deletion mutants against the model insect via normal cuticle infection, a loss of their ability to grow out of cadaver surfaces by cuticular penetration, and great reductions in total ECE and Pr1 activities critial for cuticle degradation [38,39]. Since the deletion mutants were compromised slightly in conidial germination and not affected in hyphal growth on agar plates or in submerged cultures, we consider that the reduced ECE and Pr1 activities could be responsible for a loss of their pathogenicity and likely associated with blocked secretion of those enzymes due to the disturbance of their cellular homeostasis. On the other hand, important roles of *ena1a* and *ena2b* in virulence-related cellular events are uncovered by attenuated virulence of their deletion mutants through the cuticle-bypassing infection and a correlation of attenuated virulence with reduced dimorphic transition rate in TPB mimicking insect hemolymph. Transition of multicellular hyphae to unicellular hyphal bodies after entry into host hemocoel and *vice versa* near the time of host death are critical for intrahaemocoel fungal propagation by yeast-like budding to accelerate host death from mummification and for fungal outgrowth and ultimate conidiation on cadaver surfaces respectively [44–46]. The dimorphic transition crucial for fungal virulence and infection cycle was compromised *in vivo* and *in vitro* when *ena1a* or *ena2b* lost function. This indicates a close link of either *ena1a*- or *ena2b*-mediated cellular homeostasis to dimorphic transition. Previously, abolished host infection through cuticular penetration and greatly attenuated virulence through injection were associated with vacuolar acidification and severe growth and development defects in the absence of *nhx1* [36]. In this study, none of the deletion mutants was defective in hyphal growth and development, suggesting a link of attenuated virulence to some other cellular events. We speculate that exposure to high osmolarity in insect hemolymph and oxidative stress from insect immunity defense [5] could have retarded the germination of injected mutant conidia and the development of hyphal bodies from their germ tubes since both Δ*ena1a* and Δ*ena2b* were sensitive to such stress cues. Therefore, their virulence was largely attenuated via the cuticle-bypassing infection.

In conclusion, *ena1a* and *ena2b* are essential for homeostatic processes of vacuolar H^+^, cytosolic Na^+^/K^+^ and extracellular H^+^ in *B. bassiana* and hence important for the fungal adaptation to host and environment. This is unveiled by cytosolic Na^+^/K^+^ levels increased at the time of vacuolar acidification in Δ*ena1a* or Δ*ena2b* but rarely increased when vacuolar acidification was not impeded by singular deletions of other nonfunctional ENA or NK genes. These findings provide novel insight into a link of vacuolar H^+^ level to functional ENA-mediated cellular Na^+^/K^+^ homeostasis in a filamentous fungal pathogen.

## Materials and methods

### Bioinformatic analysis of ENA and NK ATPases

The ENA1 and ENA2 sequences of *S. cerevisiae* were used as queries to search through the genomic database of *B. bassiana* under the NCBI accession NL_ADAH00000000 [29] at http://blast.ncbi.nlm.nih.gov/Blast.cgi/. The sequence features of seven homologues located in the fungal genome were individually analyzed at https://www.ncbi.nlm.nih.gov/Structure/ to reveal possible differences one another, followed by phylogenetic analysis of these homologues, the used queries and the counterparts found in other entomopathogenic and non-entomopathogenic fungi with a neighbor-joining method in the online software MEGA7 at http://www.megasoftware.net/. Molecular features of five ENA and two NK ATPases recognized through the analyses were further analyzed, including transmembrane motifs predicted at http://www.cbs.dtu.dk/services/TMHMM/, subcellular localization predicted at https://wolfpsort.hgc.jp/, and molecular size and isoelectric point estimated at https://web.expasy.org/compute_pi/.

### Transcriptional profiling of ENA and NK genes

For insight into transcriptional responses of all ENA and NK genes to ambient pH and Na^+^/K^+^ cues, the WT cultures were prepared by incubating 100 aliquots of a 10^6^ conidia/ml suspension in CDB (3% sucrose, 0.3% NaNO_3_, 0.1% K_2_HPO_4_, 0.05% KCl, 0.05% MgSO_4_ and 0.001% FeSO_4_) at optimal 25°C for 3 days on a shaking bed (150 rpm). Initial pH of CDB was adjusted to 6.0, 7.0, 8.0 and 9.0 respectively or to 7.0 after NaCl or KCl was added to the final concentration of 0.4 M. Total RNAs were extracted from the different cultures with RNAiso^TM^ Plus Reagent (TaKaRa, Dalian, China) and reversely transcribed into cDNAs with PrimeScript^RT^ reagent kit (TaKaRa). Three cDNA samples derived from the cultures of each treatment were used as templates to assess transcript levels of each target gene via real-time quantitative PCR (qPCR) with paired primers (Supplementary Table S2) under the action of SYBR_Premix ExTaq^TM^ (TaKaRa). Transcripts were normalized using γ-actin gene transcript as a reference. A threshold-cycle (2^−ΔΔCt^) method was used to compute relative transcript levels of each gene in the WT cultures under acidic, alkaline and Na^+^/K^+^ stresses with respect to the standard at pH 7.0.

### Targeted gene deletion and complementation

Our plasmids p0380-bar and p0380-sur-gateway constructed previously [47] were used as backbones to create targeted gene deletion and complemented mutants. Briefly, paired primers in Supplementary Table S2 were used to amplify 5′and 3′ coding/flanking fragments of each target gene from the genomic DNA of the WT strain under the action of high-fidelity LA-Taq enzyme. The amplified fragments were inserted into appropriate cleavage sites of p0380-bar after digestion with specific restriction endonucleases, yielding the vectors p0380-5′*x*-bar-3′*x* (*x* = *ena1a, ena1b, ena1c, ena2a, ena2b, nk1* and *nk2* respectively) for targeted gene deletion. Next, the full-length coding/flanking sequence of each gene was amplified from the WT DNA with paired primers and inserted into p0380-sur-gateway to exchange for the gateway fragment under the action of Gateway® BP Clonase^TM^ II enzyme, forming the complementary vectors p0380-sur-*x*. After verification via PCR and digestion with restriction enzymes, the method of *Agrobacterium*-mediated transformation was used to transform each deletion plasmid into the WT strain for homologous recombination and to ectopically integrate each complementary vector into an identified deletion mutant of each gene for targeted gene complementation. Putative deletion and complementation mutants were screened by the *bar* resistance to phosphinothricin (200 μg/ml) and the *sur* resistance to chlorimuron ethyl (10 μg/ml), respectively. All putative mutants were grown for 3 days on the plates of SDAY (4% glucose, 1% peptone and 1.5% agar plus 1% yeast extract), followed by DNA extraction and identification through PCR and Southern blot hybridization with paired primers and amplified probes (Supplementary Table S2). Pairs of positive *ena* and *nk* mutants were evaluated in parallel with the parental WT strain in the following experiments of three independent replicates unless specified otherwise.

### Assays for cellular cation homeostasis

For all deletion mutants and control strains, 50 ml aliquots of a 10^6^ conidia/ml CDB with an unadjusted pH of ~7.5 were incubated on the shaking bed for 5 days at 25°C. From 24-h incubation onwards, pH value was daily quantified as an index of extracellular H^+^ level from each of the cultures using an electronic pH meter.

Since the measured pH declined gradually in the cultures of all control strains from day 3 onwards, vacuolar pH was assessed as an index of intracellular H^+^ level from the 3-day-old CDB cultures of each strain as described previously [36,48]. Briefly, hyphae collected from each culture were washed repeatedly with 50 mM PBS (pH 7.4) and resuspended in the PBS, followed by adding 2.5 mM of fluorescent dye BCECF-AM [2,7-bis(2-carboxyethyl)-5,6-carboxyfluorescein-acetoxymethyl ester; Molecular Probes) to the suspension for final 50 μM. The suspension was incubated on the shaking bed for 30 min at 25°C, followed by collection of stained hyphae by centrifugation. The collected hyphae were rinsed twice with PBS for removal of dye residue. The fluorescence intensity was read from each of 15 vacuoles in each of three view fields of stained hyphae at the excitation/emission wavelengths of 450/535 and 490/535 nm under a confocal microscope respectively. The ratio of the two readings at 450/535 and 490/535 nm was used to estimate vacuolar pH by interpolating the ratio into the calibration equation *y* = 0.344*x* – 1.263 (r^2^ = 0.994), in which *x* and *y* denote vacuolar pH and the ratio respectively. The equation was established by equilibrating hyphae for 60 min in the respective buffers of 50 mM MES [2-(N-morpholino) ethanesulphonic acid] from pH 5.0 to 5.5 and 50 mM HEPES {2-[4-(2-Hydroxyethyl)-1-piperazinyl] ethanesulfonic acid} from pH 6.0 to 6.5 (Supplementary Fig. S4). Each buffer contained 50 mM KCl, 50 mM NaCl, 0.2 M ammonium acetate and 10 mM sodium azide.

For insight into a link of extracellular and vacuolar pH (H^+^) levels to cytosolic Na^+^/K^+^ levels, the Na^+^- and K^+^-specific fluorescence probes SBFI-AM and PBFI-AM (Nanjing KeyGen Biotech Co., Najing, China) were used to quantify cytosolic Na^+^ and K^+^ levels from hyphal cells of each strain, respectively. Following the manufacturer’s guide, briefly, 10 mM of each probe in DMSO (dimethyl sulfoxide) was mixed in equal volume with 25% solution of the non-ion surfactant Pluronic F-127 (Beyotime, Shanghai, China) in DMSO, forming a surfactant-containing mixture enabling to increase membrane permeability for rapid assessment of Na^+^ or K^+^ flux in cells. Aliquots of 50 ml 10^7^ conidia/ml CDB were incubated 24 h on the shaking bed at 25°C. Cells collected from each culture by centrifugation were resuspended into fresh CDB with initial pH adjusted to 5.0, 7.0 and 9.0 respectively, followed by a 30-min incubation. The resultant cells were collected, washed three times with PBS (pH 7.4) and resuspended in 1 ml of PBS containing the mixture and 10 μM of each probe. The suspension was incubated for 30 min at 37°C, followed by collection of stained cells. The stained cells were washed twice in PBS for removal of dye residue and resuspended in 1 ml of PBS. Subsequently, 100 μl aliquots of each suspension were added to a 96-well plate, and fluorescent intensity (FI) was quantified from each well at the alternating excitation wavelengths of 340/380 nm with fixed emission wavelength of 500 nm in the microplate reader SpectraMax M5/M5e (Molecular Device, Shanghai, China). The ratio of FI_340_ over FI_380_ was calculated as relative Na^+^ or K^+^ content (flux) in the stained cells. The experiment of each strain included three independent cell cultures as replicates.

### Subcellular localization of ENA1a and ENA2b

The vector pAN52-C-gfp-bar with the C cassette 5′-*Pme*I-*Spe*I-*Eco*RV-*Eco*RI-*Bam*HI-3′ under the control of P*tef1*, a promoter of the homologous gene encoding translation elongation factor 1 alpha [49], was used to clarify transmembrane and vacuolar activities of ENA1a and ENA2b via subcellular localization. Briefly, the vector was digested with *Xma*I/*Bam*HI. The open reading frame (ORF) of *ena1a* or *ena2b* was amplified from the WT cDNA with paired primers (Supplementary Table S2) and inserted into the N-terminus of *gfp* (green fluorescence protein gene) in the linearized vector using one-step cloning kit (Vazyme, Nanjin, China). The resultant pAN52-*x*::gfp-bar (*x* = *ena1a* or *ena2b*) was integrated into the WT strain via *Agrobacterium*-mediated transformation. Putative transformants were screened by the *bar* resistance to phosphinothricin (200 μg/ml) and examined under a fluorescence microscope. A transformant from each transformation was chosen with desired green signal under a fluorescence microscope and incubated on SDAY for conidiation. The conidia were suspended in SDBY (i.e., agar-free SDAY) and incubated at 25°C for 24 to 72 h on the shaking bed. Hyphal samples collected from each culture were stained with FM4-64 (Invitrogen, Shanghai, China) and visualized for subcellular localization of GFP-tagged ENA1a and ENA2b fusion proteins through LSCM.

### Assays for radial growth and stress response

For all strains, 1 μl aliquots of a 10^6^ conidial/m suspension were spotted centrally on the plates of SDAY and CDA (i.e., CDB plus 1.5% agar) for normal growth and on the plates of pH-unadjusted CZA alone (control) or supplemented with each of the following chemical agents for stress response. The agents added to the unadjusted CDA included: (a) 0.4, 0.7 and 1.0 M of NaCl or KCl for responses to Na^+^/K^+^ gradient; (b) LiCl (10 mM), ZnSO_4_ (3 mM), MnSO_4_ (3 mM), CuSO_4_ (2 mM) and FeCl_3_ (1 mM) for responses to different metal cations; and (c) H_2_O_2_ (2 mM), menadione (0.02 mM), Congo red (3 μg/ml), calcofluor white (10 μg/ml) and sorbitol (1 M) for responses to stresses of oxidation, cell wall perturbation and high non-cation osmolarity respectively. Moreover, the CDA plates were supplemented with 0.4 M NaCl, 0.4 M KCl and 10 mM LiCl, followed by adjusting initial pH to 5.0, 7.0 and 9.0 for cellular responses to Na^+^, K^+^ and Li^+^ under acidic, neutral and alkali conditions. The cation-free plates at pH 7.0 were used as a control. After a 7-day incubation at the optimal regime of 25°C in a light/dark (L:D) cycle of 12:12 h, the diameter of each colony was estimated as an growth index with two measurements taken perpendicular to each other across the center. Relative growth inhibition (%) of each strain under each stress was used as a stress-responsive index and computed as (*D*_c_–*D*_t_)/*D*_c_×100, where *D*_c_ and *D*_t_ denote the colony diameters of the control and each stress treatment respectively.

### Assays for conidial yield, germination and UVB resistance

To assess conidial yields of all strains, 100 μl aliquots of a 10^7^ conidia/ml suspension were spread on SDAY plates (9 cm diameter), followed by a 9-day incubation at the optimal regime. Three plugs (0.5 mm diameter) were taken from each of the plate cultures using a cork borer. The conidia of each plug were released into 1 ml of 0.05% Tween 80 through a supersonic vibration of ~10 min. The conidial concentration in the resultant suspension was assessed using a hemocytometer and converted to the number of conidia per unit area (cm^2^) of plate culture. The suspension of each strain was diluted to a final concentration of 10^6^ conidia/ml and every 100 μl was spread onto an agar plate for incubation at 25°C, followed by estimation of germination rate with microscopic counts at 2-h interval. GT_50_ (h) required for 50% germination of each strain at 25ºC was estimated through modeling analysis of each time-germination trend. In addition, conidial resistance to UV-B irradiation was estimated as LD_50_ (J/cm^2^) from declining trend of conidial viability over gradient UVB doses as described previously [50].

### Assays for pathogenicity, virulence and associated cellular events

Standardized bioassays were carried out for pathogenicity and virulence of each strain again the fifth-instar larvae of *G. mellonella* through two infection modes. Briefly, three groups of ~35 larvae were immersed for 10 s in 40 ml aliquots of a 10^7^ conidia/ml suspension for normal infection through cuticular penetration. Alternatively, 5 μl of a 10^5^ conidia/ml suspension was injected into the hemocoel of each larva in each group for cuticle-bypassing infection. Groups of larvae immersed or injected with 0.02% Tween 80 for preparation of conidial suspension were used as controls in the corresponding assays. All groups inoculated by topical application (immersion) or intrahaemocoel injection were maintained at 25°C for 10 days and examined daily for survival/mortality records. Median lethal time (LT_50_) of each strain against the model insect was estimated as a virulence index through probit analysis of the time-mortality trend in each group.

To reveal the development of hyphal bodies *in vivo* after dimorphic transition, hemolymph samples were taken from the larvae surviving different periods after injection and examined for the presence/absence and abundance of hyphal bodies in the samples as described previously [40,41]. The larvae mummified by injected conidia were maintained at 25°C to show whether intrahaemocoel hyphal bocides turn into hyphae to penetrate through the insect cuticle for outgrowth on cadaver surfaces. The observed *in vivo* cellular events critical for fungal pathogenicity and virulence were examined further in the following *in vitro* experiments. First, 50 ml aliquots of a 10^6^ conidia/ml suspension in CDB containing 0.3% bovine serum albumin (BSA) as sole nitrogen source and enzyme inducer were incubated 3 days on the shaking bed at 25ºC. The supernatants of the resultant cultures were used as crude extracts to quantify total ECE and Pr1 activities as described elsewhere [51,52]. One unit of enzyme activity was defined as the amount of ECE or Pr1 required for an increase in the optical density at 410 or 440 nm (OD_410_ or OD_440_) of 0.01 after a 1-h reaction of each supernatant versus a control, and total activity was expressed as the number of units per ml supernatant. Next, biomass levels (mg/ml) and blastospore concentrations were quantified from the 3-day-old submerged cultures initiated with 50 ml aliquots of a 10^6^ conidia/ml TPB, i.e., CZB amended with the sole carbon source of 3% trehalose and the sole nitrogen source of 0.5% peptone to mimic insect hemolymph. Dimorphic transition rate (no. blastospores/mg biomass) of each strain was computed from the two parameters measured from the *in vitro* TPB cultures and used as a reference to a speed of intrahaemocoel fungal proliferation and host death from mummification.

### Statistical analysis

All experimental data were subjected to one-factor (strain) analysis of variance, followed by Tukey’s honestly significant difference (HSD) test for a comparison of phenotypic means between deletion mutants and control strains.

## Supplementary Material

Supplemental Material
