# Supplementary material for: P-type Na+/K+ ATPases essential and nonessential for cellular homeostasis and insect pathogenicity of Beauveria bassiana
Source: Virulence. 2020 Oct 24;11(1):1415–31. doi: 10.1080/21505594.2020.1836903 (PMC7588218; doi:10.1080/21505594.2020.1836903)
Supplement: Supplemental Material [file KVIR_A_1836903_SM5071.docx]

**Supplementary Material**

**P-type Na^+^/K^+^ ATPases essential and nonessential for cellular homeostasis and insect pathogenicity of *Beauveria bassiana***

Ya-Ni Mou, Ben-Jie Gao, Kang Ren, Sen-Miao Tong, Sheng-Hua Ying, and Ming-Guang Feng*

**MOE Laboratory of Biosystems Homeostasis & Protection,** Institute of Microbiology, College of Life Sciences, Zhejiang University, Hangzhou, Zhejiang, 310058, China

*Correspondence should be addressed to Ming-Guang Feng, mgfeng@zju.edu.cn

**Table S1.** Predicted molecular properties of ENA and NK ATPases in *B. bassiana*.

| ENA/NK ATPase | Gene  length  (bp) | No. introns | cDNA length  (bp) | Molecular size  (kD) | Isoelectic  point | Subcellular  localization Predicted* | Sequence identity (%) to one another | | | | | | |
| --- | --- | --- | --- | --- | --- | --- | --- | --- | --- | --- | --- | --- | --- |
|  |  |  |  |  |  |  | ENA1a | ENA1b | ENA1c | ENA2a | ENA2b | NK1 | NK2 |
| ENA1a | 3757 | 3 | 3249 | 119.2 | 6.07 | PM | 100 |  |  |  |  |  |  |
| ENA1b | 3685 | 3 | 3255 | 119.6 | 6.73 | PM | 82 | 100 |  |  |  |  |  |
| ENA1c | 3416 | 3 | 3198 | 115.4 | 5.46 | PM | 56 | 57 | 100 |  |  |  |  |
| ENA2a | 3529 | 3 | 3354 | 122.2 | 7.78 | PM | 47 | 47 | 46 | 100 |  |  |  |
| ENA2b | 3176 | 2 | 3072 | 110.1 | 5.37 | PM | 46 | 46 | 44 | 42 | 100 |  |  |
| NK1 | 3324 | 0 | 3324 | 122.3 | 6.38 | PM | 26 | 26 | 28 | 27 | 29 | 100 |  |
| NK2 | 3643 | 5 | 3327 | 121.8 | 6.58 | PM | 26 | 26 | 26 | 26 | 27 | 42 | 100 |

* All ENA and NK ATPases are predicted to localize to the plasma membrane (PM) at http://www.cbs.dtu.dk/services/TMHMM/. Predicted tansmembrane regions include residues 76-98, 102-121, 303-320, 330-352, 794-816, 826-848, 877-899, 919-941, 970-992 and 1002-1023 for ENA1a; residues 78-100, 104-123, 306-328, 333-355, 797-819; 829-851, 880-902, 931-953, 974-996 and 1006-1024 for ENA1b; residues 86-108, 112-129, 281-298, 302-324, 333-355, 817-836, 843-865, 899-921, 942-964, 984-1006 and 1013-1035 for ENA1c; residues 61-78, 82-101, 289-311, 326-348, 818-840, 850-872, 897-919, 942-964, 993-1011 and 1026-1048 ENA2a; residues 97-119, 123-142, 313-330, 340-362, 772-794, 809-826, 855-877, 897-919, 949-971 and 981-999 for ENA2b; Residues 171-193, 203-225, 318-340, 369-391, 404-426, 889-911, 932-954, 995-1017, 1037-1059 and 1063-1085 for NK1; and residues 150-172, 182-204, 344-366, 376-395, 866-888, 893-915, 936-958, 995-1017, 1038-1060 and 1065-1087 for NK2, respectively.

**Table S2.** Paired primers used for manipulation of ENA and NK genes in *B. basssiana*.

| Primers | Paired sequences (5′−3′) ^a^ | Purpose ^b^ |
| --- | --- | --- |
| ENA1a-F/R | CAATCACAAACACCTTCAAAATGGGCCAGGAAAAGGAGG/TCCTCGCCCTTGCTCACCAT TACAGAAATCTCATCGCTGCG | Cloning *ena1a* cDNA (3246 bp) for fusion to *gfp* |
| ENA2b-F/R | CAATCACAAACACCTTCAAAATGGGAGAAACCGCCGCTGTTATTA/TCCTCGCCCTTGCTCACCAT TGCAGGCGAAAGAGCAGCCG | Cloning *ena2b* cDNA (3069 bp) for fusion to *gfp* |
| upENA1a-F/R | TGGGCCCGGCGCGCCGAATTCGATGTGGACGAAGAAGGC / TGGCTGCAGGTCGAC GGATCCAAGGGACGTTGCGAAGAT | Cloning 3′ *ena1a* (1653 bp) |
| dnENA1a-F/R | GACCCATGGCTCGAGTCTAGACAACGCCCTCACTCTGGT / GTGGCTAGCGTTAACAC TAGTTGGTTTGGCACGCCTACT | Cloning 5′ *ena1a* (1317 bp) |
| flENA1a-F/R | GGGGACAAGTTTGTACAAAAAAGCAGGCTCCGAAAGAAATAACCAACTC / GGGGA CCACTTTGTACAAGAAAGCTGGGTCAACAATGGCACAACAGG | Cloning full-length *ena1a* |
| upENA1b-F/R | TGGGCCCGGCGCGCCGAATTCTTCTCGGCACAGCCTCCCT / TGGCTGCAGGTCGACG GATCCGTCGCTGAATCGAATTGTTTGG | Cloning 5′ *ena1b* (1646 bp) |
| dnENA1b-F/R | GACCCATGGCTCGAGTCTAGACCATGAAGTTGGCCTGAC / GGTGGTGGTGGCTAGC GTTAACGACCTCCTGGCTGTTGCT | Cloning 3′ *ena1b* (1242 bp) |
| flENA1b-F/R | GGGGACAAGTTTGTACAAAAAAGCAGGCTAGTTTGCTGGCTGCTTAT / GGGGACCA CTTTGTACAAGAAAGCTGGGTATTCTACGTGGCTCATCG | Cloning full-length *ena1b* |
| upENA1c-F/R | TGGGCCCGGCGCGCCGAATTCTACCAGCGAGACCACATAC / TGGCTGCAGGTCGACG GATCCAAGGAATAAAGAGCCAGAAC | Cloning 5′ *ena1c* (1437 bp) |
| dnENA1c-F/R | GACCCATGGCTCGAGTCTAGACTTGACGATATGGGCTAACA / GTGGCTAGCGTTAAC ACTAGTAAAGTCGGAGGCGGAGAT | Cloning 3′ *ena1c* (1704 bp) |
| flENA1c-F/R | GGGGACAAGTTTGTACAAAAAAGCAGGCTTGACGCGACATCATAGACC / GGGGACC ACTTTGTACAAGAAAGCTGGGTACGAAGCCGAGCAAAGTA | Cloning full-length *ena1c* |
| upENA2a-F/R | TGGGCCCGGCGCGCCGAATTCCCAAGACCCAGGTAATCG / TGGCTGCAGGTCGACGG ATCCATGAAGGTGCGAAGGTGA | Cloning 3′ *ENA2a* (1828 bp) |
| dnENA2a-F/R | GACCCATGGCTCGAGTCTAGACTTCGGCATCAAAGATTACA / GTGGCTAGCGTTAACA CTAGTGCTGGCTCAGCTTCACTC | Cloning 5′ *ena2a* (1263 bp) |
| flENA2a-F/R | GGGGACAAGTTTGTACAAAAAAGCAGGCTTTAATCAAGCACCAAGTCCA / GGGGACC ACTTTGTACAAGAAAGCTGGGTGAGAAATCACGCATCAGAAA | Cloning full-length *ena2a* |
| upENA2b-F/R | TGGGCCCGGCGCGCCGAATTCTCCCTCATTATTCCAGACA / TGGCTGCAGGTCGACGG ATCCAGGCGAAGGATTTAGTTT | Cloning 3′ *ena2b* (1573 bp) |
| dnENA2b-F/R | GACCCATGGCTCGAGTCTAGATGGTTTGACTACCGAAGAGG / GTGGCTAGCGTTAAC ACTAGTCGGGATTCACATTTAGCG | Cloning 5′ *ena2b* (1647 bp) |
| flENA2b-F/R | GGGGACAAGTTTGTACAAAAAAGCAGGCTGTTTGCCAACGACTTCATC / GGGGACCA CTTTGTACAAGAAAGCTGGGTGACGGTTTCAACTTTCAGG | Cloning full-length *ena2b* |
| upNK1-F/R | TGGGCCCGGCGCGCCGAATTCGAGACGACCGAATGAAAGC / TGGCTGCAGGTCGAC GGATCCGACGAGGCAGGGAGAATG | Cloning 5′ *nk1* (1323 bp) |
| dnNK1-F/R | GACCCATGGCTCGAGTCTAGAGAGGCTACCGAGCGAGACA / GTGGCTAGCGTTAACA CTAGTGGAAGGGCAATGGTGGAG | Cloning 3′ *nk1* (1532 bp) |
| flNK1-F/R | GGGGACAAGTTTGTACAAAAAAGCAGGCTATTGCCCAGGCGATAAGA / GGGGACCA CTTTGTACAAGAAAGCTGGGTGCGGTTTCACGAGGATTG | Cloning full-length *nk1* |
| upNK2-F/R | TGGGCCCGGCGCGCCGAATTCACCCTGAAACCGTGGTGA / TGGCTGCAGGTCGACGG ATCCGAGACAGTTGGACGTGTAAGT | Cloning 3′ *nk2* (1859 bp) |
| dnNK2-F/R | GACCCATGGCTCGAGTCTAGAAGCCGCACCAATTCCACT / GTGGCTAGCGTTAACACT AGTAGCCCACATTACCATCAGC | Cloning 5′ *nk2* (1655 bp) |
| flNK2-F/R | GGGGACAAGTTTGTACAAAAAAGCAGGCTCGAGTCGGTCAACGTCAT / GGGGACCA CTTTGTACAAGAAAGCTGGGTGCAAACAGCCTCCCAAAA | Cloning full-length *nk2* |
| pENA1a-F/R | CAGCGTATGGTTGCCAGGTT / ATGGGCGGAGTGGTAGGTC | PCR detecting *ena1a* |
| pENA1b-F/R | CGACAGATGGAAGTGGCTTAT / ACTCGTTGCGACCGTAGA | PCR detecting *ena1b* |

**Table S2** (continued)

| pENA1c-F/R | ACAAACGGGTCAATCTGG / CGAGGATACCGCCTTCTAC | PCR detecting *ena1c* |
| --- | --- | --- |
| pENA2a-F/R | ATCGTCAGCGGGAAACAC / TTGCCGTCACGGAGAACC | PCR detecting *ena2a* |
| pENA2b-F/R | CTGTCCGTGACCTAACTGG / TGGCACCTTTGATGGTATTG | PCR detecting *ena2b* |
| pNK1-F/R | ACAAGCCGAAACAATGGG / TGAGGTTGGCAAAGTAGTCC | PCR detecting *nk1* |
| pNK2-F/R | CCTCTTTCGCCTGGTTTC / CGCTCTTTGCCTTTGGAC | PCR detecting *nk2* |
| sENA1a-F/R | AAAAGCGGTGGTGGTTAC / ACGGAGGTTAGTTGAGAAGT | Southern probe of *ena1a* |
| sENA1b-F/R | CGACCCAGCCTGAAGTAG / GGGAAGCCCAGACAAGTA | Southern probe of *ena1b* |
| sENA1c-F/R | CGTTTGGTAGGCTTCGTT / GGCAATAGTTGTTCGGTTT | Southern probe of *ena1c* |
| sENA2a-F/R | GAGACGAGCGAAGCAACA / TGCCAAGCAGGAATAACG | Southern probe of *ena2a* |
| sENA2b-F/R | CATTGTCGTCGGGTGAGT / TGCTGATGGCAACGGATT | Southern probe of *ena2b* |
| sNK1-F/R | CAGGACAAGCCGAAACAA / CAGAGCAAGAAGAGGAGGG | Southern probe of *nk1* |
| sNK2-F/R | TCTTGGTGACCTGGAATGG/GCCAGCAGAACAATAGCG | Southern probe of *nk2* |
| qENA1a-F/R | TCTGGATGTCTGCACTTTGC / GAGAGCAAACCAGGTCAAGC | qPCR detecting *ena1a* |
| qENA1b-F/R | CTACCAGCGAGCCTTTCAAC / AGCAATTTCAGTGGGGTCAC | qPCR detecting *ena1b* |
| qENA1c-F/R | CTTTTCGGTAACGGTCGTGT / GACGAAAGTAGGCACGCTTC | qPCR detecting *ena1c* |
| qENA2a-F/R | CCGGAGTTGAACACCAAGTT / CTTGAAGCTCGACATGGACA | qPCR detecting *ena2a* |
| qENA2b-F/R | CGCCGAAAGAAGAAAGACAC / CACAGATGCCGTAGAGCAAA | qPCR detecting *ena2b* |
| qNK1-F/R | AACCCAAGCAGTACCGTGTC / TTTCGCAGAGAACAATGACG | qPCR detecting *nk1* |
| qNK2-F/R | CACCGTTGTCCTGGTTCTTT / TCTTGAGCGACTTGCAGAGA | qPCR detecting *nk2* |
| actin-F/R | GGCAACATTGTCATGTCTGG / TTTGCTGGAAGGTGGATAGG | qPCR internal standard |

^a^ Underlined regions denote introduced cleavage sites of two pairs of restriction enzymes for homologous recombination of the bar-separated 5′ and 3′ fragments of *ena1b* (*Bam*HI/*Eco*RI and *Xba*I/*Hpa*I) and of *ena1a*, *ena1c*, *ena2a*, *ena2b*, *nk1* or *nk2* (*Bam*HI/*Eco*RI and *Xba*I/*Spe*I) for targeted gene deletion, the fragments for gateway exchange or the fragments for fusion to *gfp* at the *Xma*I/*Bam*HI sites.

^b^ PCR detection aimed at the fragments of 1404 bp for deleted *ena1a* vs. WT (709 bp), 1321 bp for deleted *ena1b* vs. WT (543 bp), 1316 bp for deleted *ena1c* vs. WT (848 bp), 1181 bp for deleted *ena2a* vs. WT (589 bp), 1419 bp for deleted *ena2b* vs. WT (711 bp), 1559 bp for deleted *nk1* vs. WT (821 bp) and 1491 bp for deleted *nk2* vs. WT (708 bp). The probes amplified for Southern blotting of deleted *ena1a, ena1b, ena1c, ena2a, ena2b, nk1* and *nk2* are 263, 270, 268, 311, 299, 257 and 294 bp in length respectively.


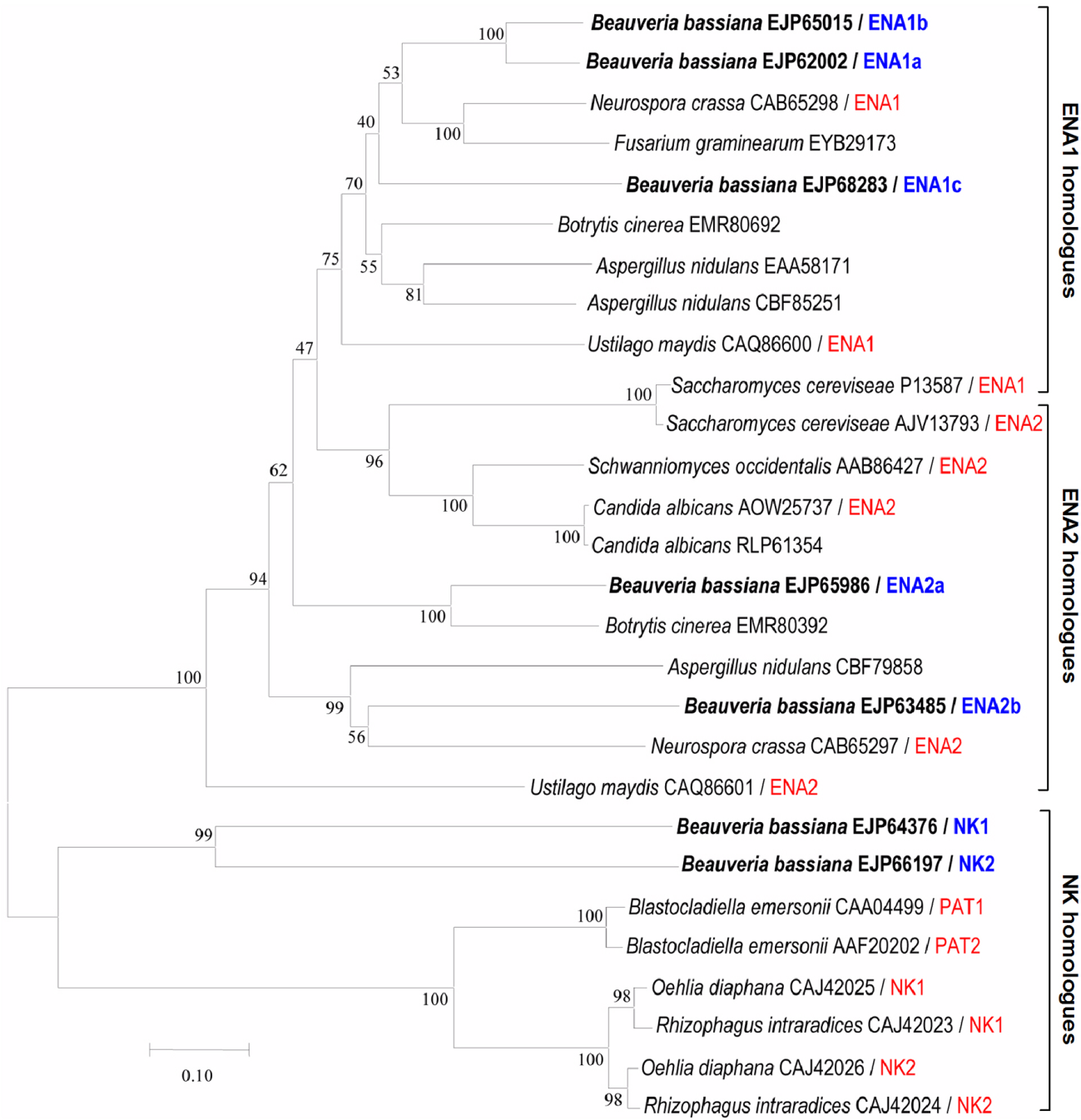


**Fig. S1.** Phylogenetic relationships of ENA1, ENA2 and NK ATPases found in yeast, yeast-like and filamentous fungi through BLASTp analysis at http://blast.ncbi.nlm.nih.gov/Blast.cgi/. The bootstrap values of 1000 replications are given at nodes. Scale: branch length proportional to genetic distance assessed with the neighbor-joining method in the software MEGA7 at http://www.megasoftware.net/. Protein names shown in red are annotated in the examined fungal genomes, and those shown in blue are renamed in this study. Note that three ENA1 and two ENA2 homologues exist in *B. bassiana*.


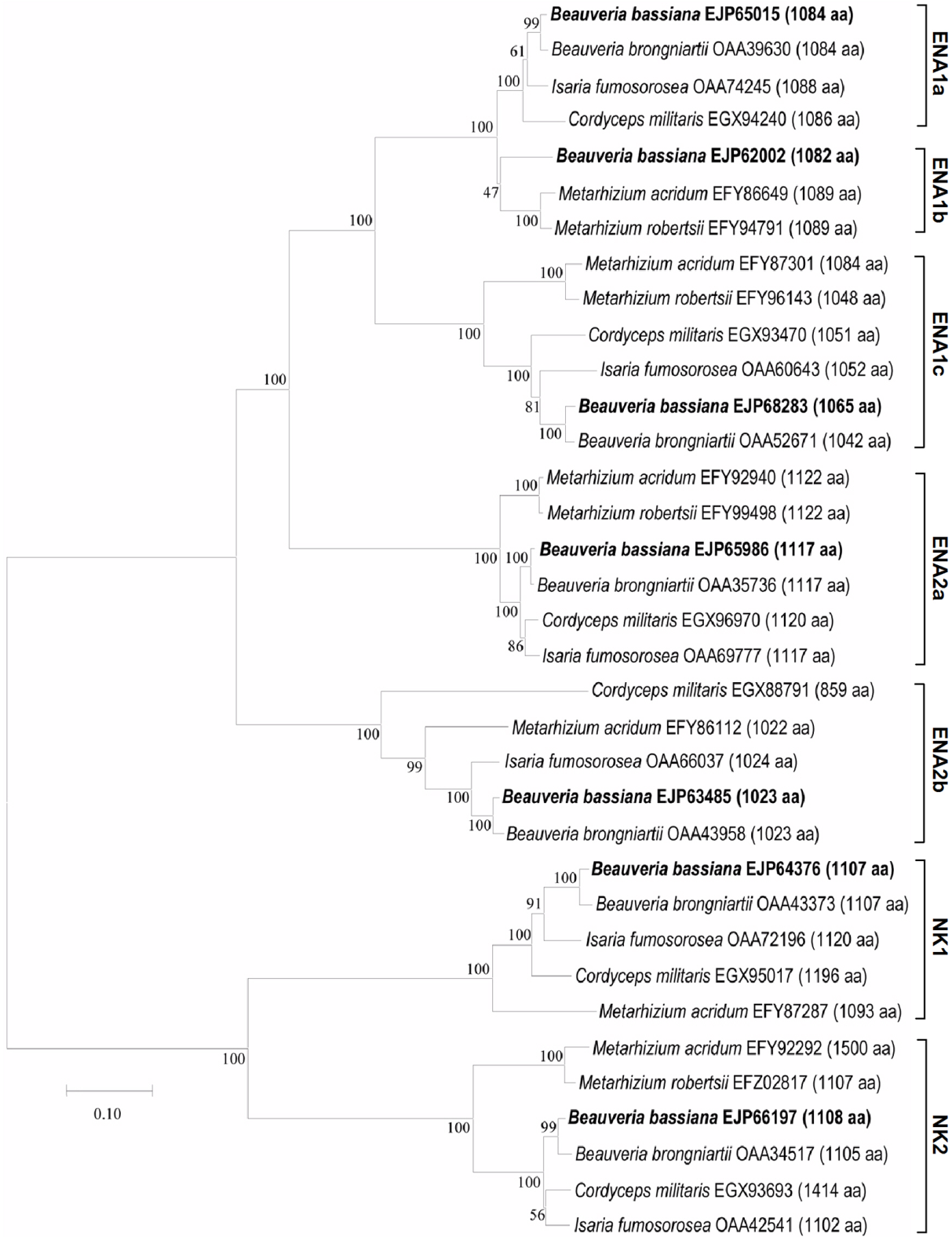


**Fig. S2.** Phylogenetic relationships of ENA1, ENA2 and NK ATPases found in *B. bassiana* and other insect- pathogenic fungi through BLASTp analysis at http://blast.ncbi.nlm.nih.gov/Blast.cgi/. The bootstrap values of 1000 replications are given at nodes. Scale: branch length proportional to genetic distance assessed with the neighbor-joining method in the software MEGA7 at http://www.megasoftware.net/. Note that multiple ENA1 and/or ENA2 homologues exist in all insect-pathogenic fungi examined.


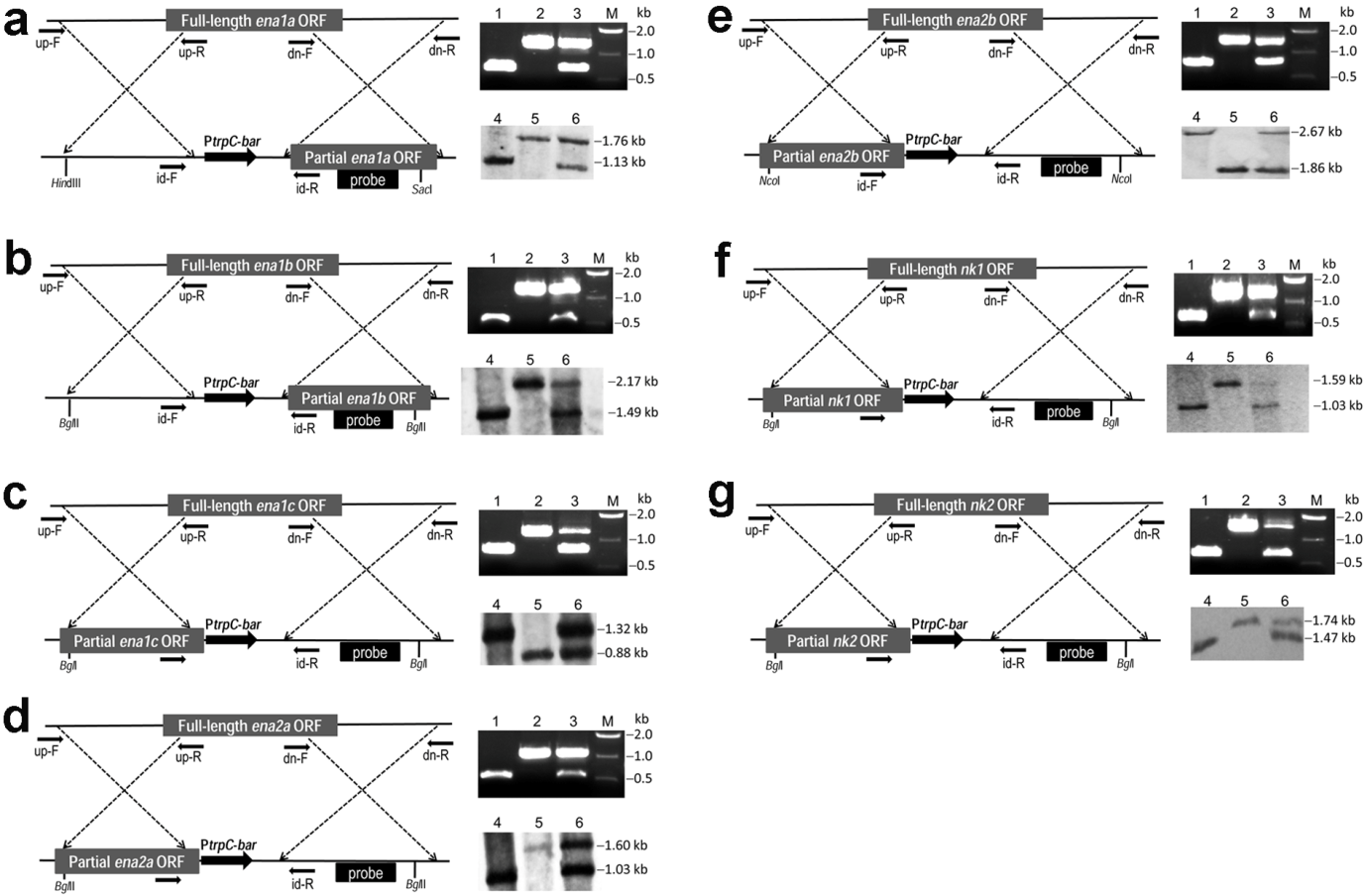


**Fig. S3.** Generation and identification of *ena* and *nk* mutants in *B. bassiana*. (**a**–**g**) Schematic diagrams for the deletion strategies of *ena1a*, *ena1b*, *ena1c*, *ena2a*, *ena2b*, *nk1* and *nk2* and their deletion and complemented mutants identified through PCR (lanes 1–3) and Southern blot (lanes 4–6) analyses with paired primers and amplified probes (Table S2), respectively. Lanes 1 and 4: parental WT. Lanes 2 and 5: deletion mutants. Lanes 3 and 6: complemented mutants. Genomic DNA for Southern blotting of each target gene was digested with appropriate enzymes at the sites indicated in the corresponding diagram.


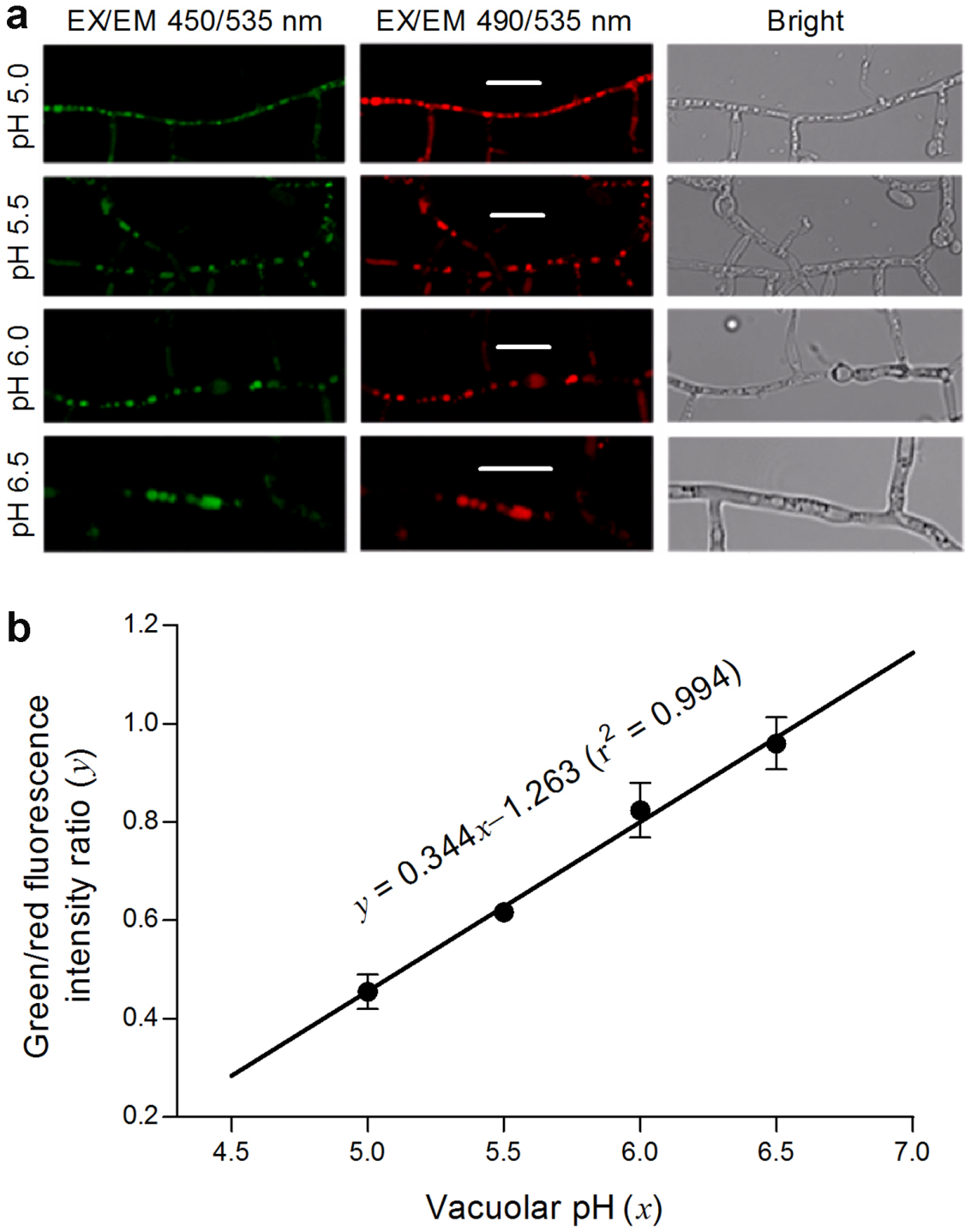


**Fig. S4.** A calibration equation established for quantification of vacuolar pH in *B. bassiana*. Hyphae collected from 3-day-old CDB cultures of a wild-type strain were stained with the fluorescent dye BCECF-AM and then equilibrated for 60 min in the buffers of 50 mM MES at pH 5.0 and 5.5 and 50 mM HEPES at pH 6.0 and 6.5 respectively, as illustrated in the upper microscopic images under a laser scanning confocal microscope. The ratios (solid circles of *y*) of the two fluorescence readings from the stained vacuoles at the excitation/emission wavelengths of 450/535 and 490/535 nm were linearly correlated to vacuolar pH (*x*), generating the calibration equation (fitted line in the lower graph) for estimation of vacuolar pH by interpolation.


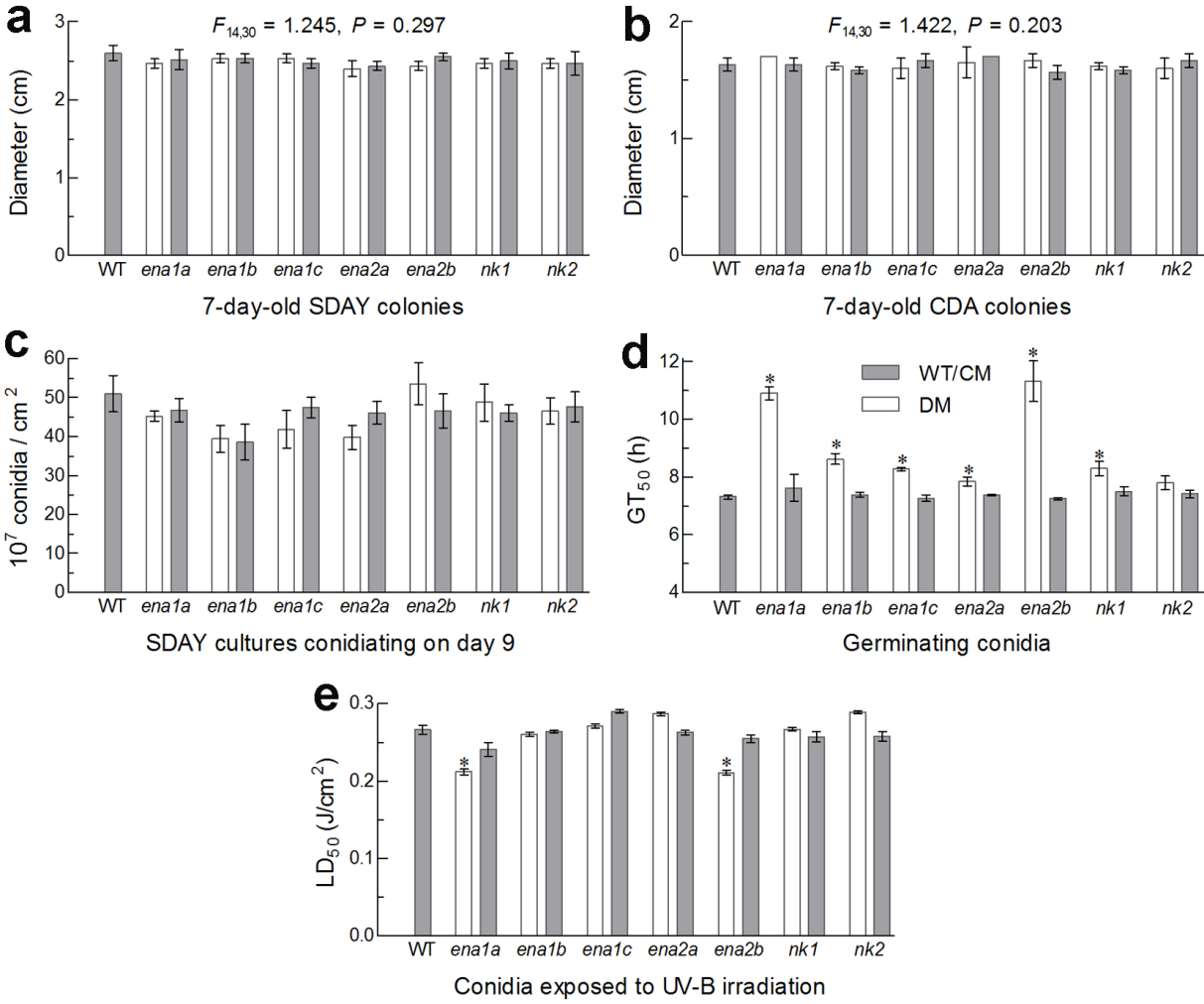


**Fig. S5.** Impacts of singular *ena* and *nk* deletions on radial growth, aerial conidiation, and conidial germination and UVB resistance in *B. bassiana*. (**a, b**) Colony diameters of WT, deletion mutants (DM) and complemented mutants (CM) incubated at optimal regime of 25°C in a light/dark cycle of 12:12 for 7 days on the respective plates of rich SDAY and minimal CDA after 1 μl of a 10^6^ conidia/ml suspension was spotted per plate for colony initiation. (**c**) Conidial yields quantified from the 9-day-old SDAY cultures initiated by spreading 100 μl of a 10^7^ conidia/ml suspension per plate and incubated at the optimal regime. (**d**) Estimates of median germination time (GT_50_) required for 50% conidial germination of tested strains at 25°C. (**e**) Estimates of median lethal dose (LD_50_) for conidial resistance of tested strains to UVB irradiation. Asterisked phenotype parameters differ significantly from those unmarked in each bar chart (Tukey's HSD, *P* < 0.05). Error bars: standard deviation of the mean from three replicates.


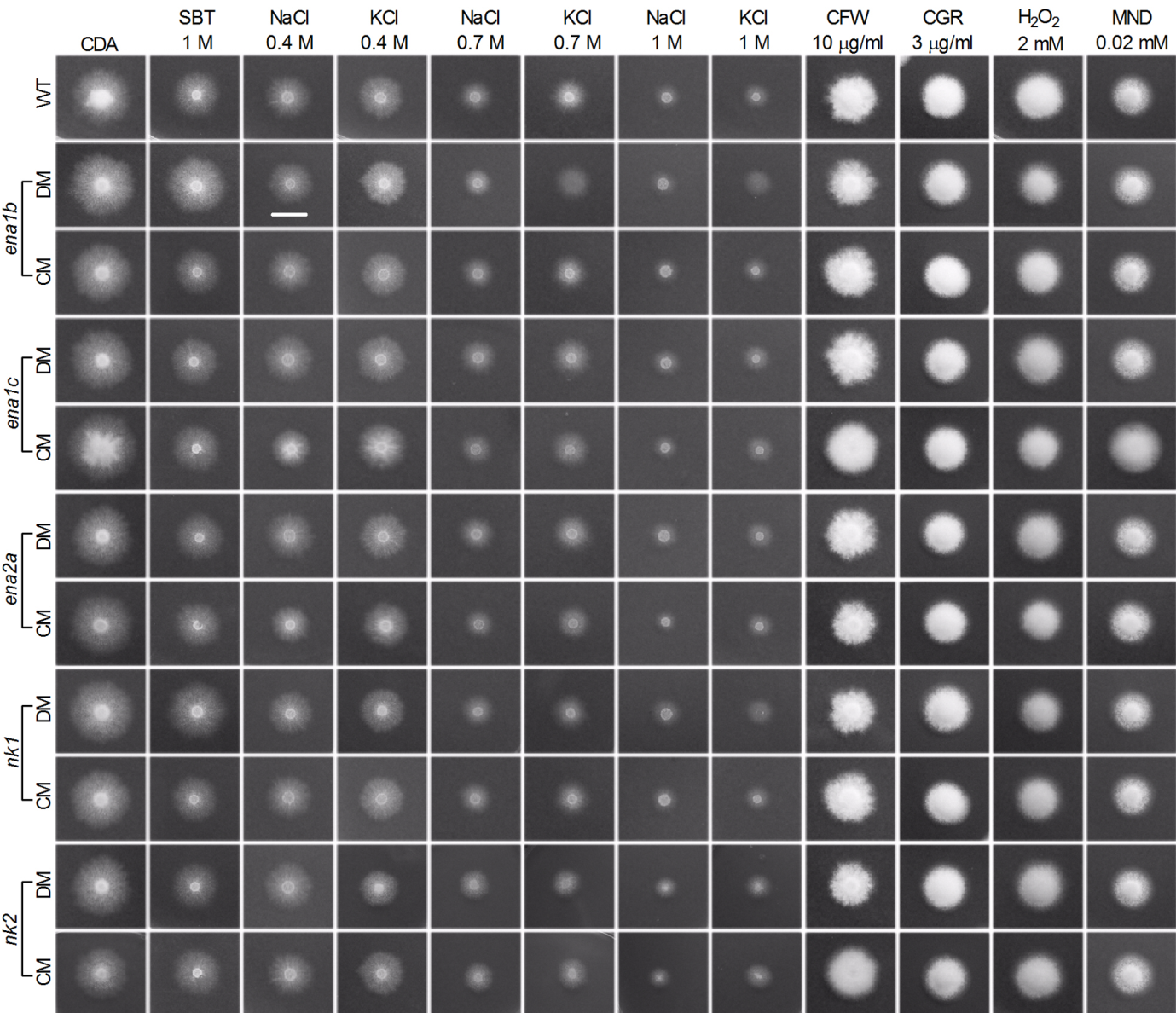


**Fig. S6.** Impacts of singular *ena* and *nk* deletions on multiple stress responses of *B. bassiana*. Images (scale bar: 10 mm) of fungal colonies were taken when WT, deletion mutants (DM) and complemented mutants (CM) were grown at optimal 25°C for 7 days on the plates of CDA alone (control) or supplemented with an indicated concentration of each stress agent (SBT, sorbitol; CFW, calcafluor white; CGR, Congo red; MND, menadione). Each colony was initiated by spotting 1 μl of a 10^6^ conidia/ml suspension.


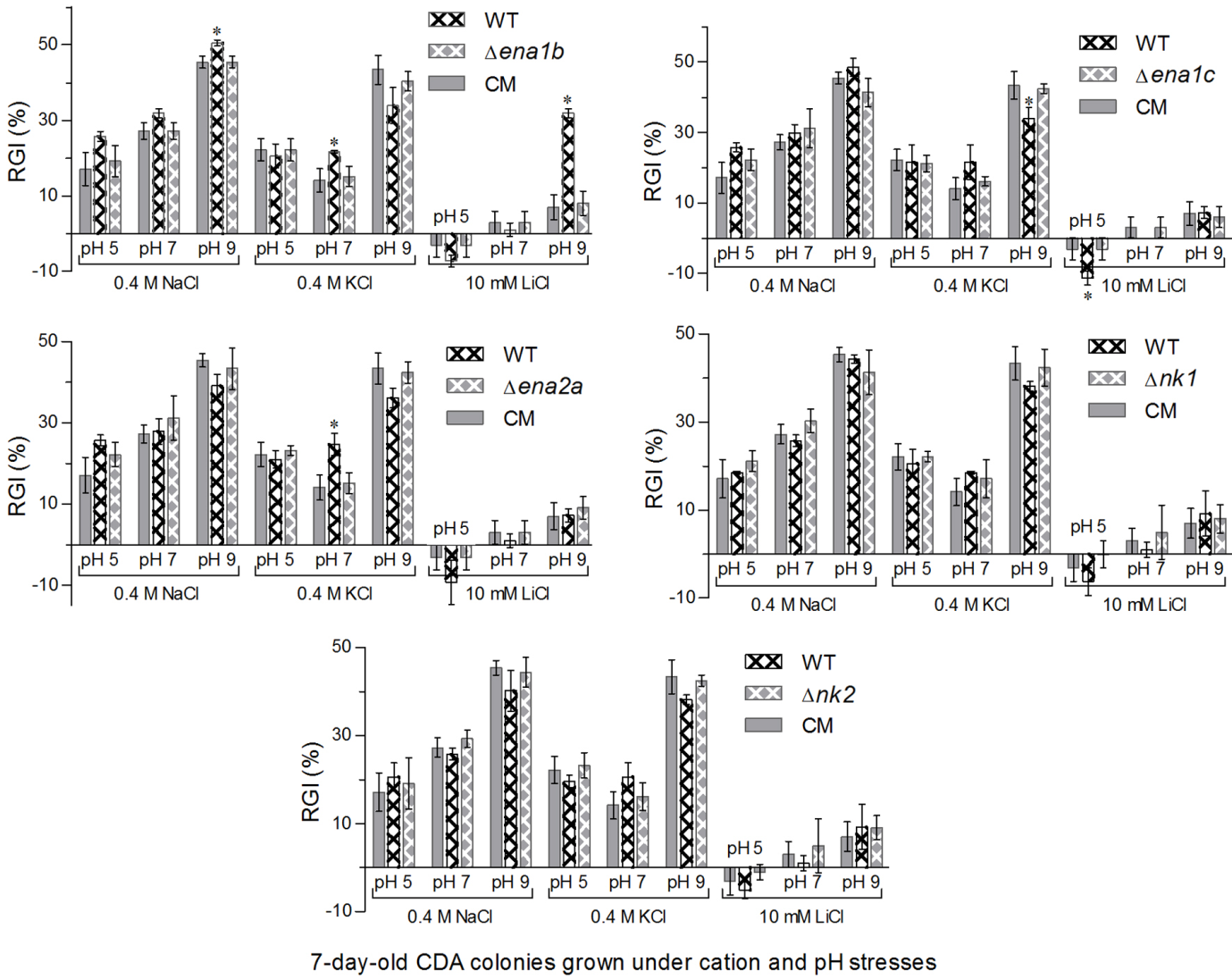


**Fig. S7.** Impacts of singular *ena* and *nk* deletions on relative growth inhibition (RGI) of *B. bassiana* on the plates of CDA supplemented with 0.4 M NaCl, 0.4 M KCl or 10 mM LiCl and then adjusted to initial pH 5.0, 7.0 and 9.0 respectively. Each colony was initiated by spotting 1 μl of a 10^6^conidia/ml suspension and incubated for 7 days at 25°C in a light/dark cycle of 12:12. Asterisked mean differed significantly from those not marked in each three-bar group (Tukey's HSD, *P* < 0.05). Error bars: standard deviation of the mean from three replicates.


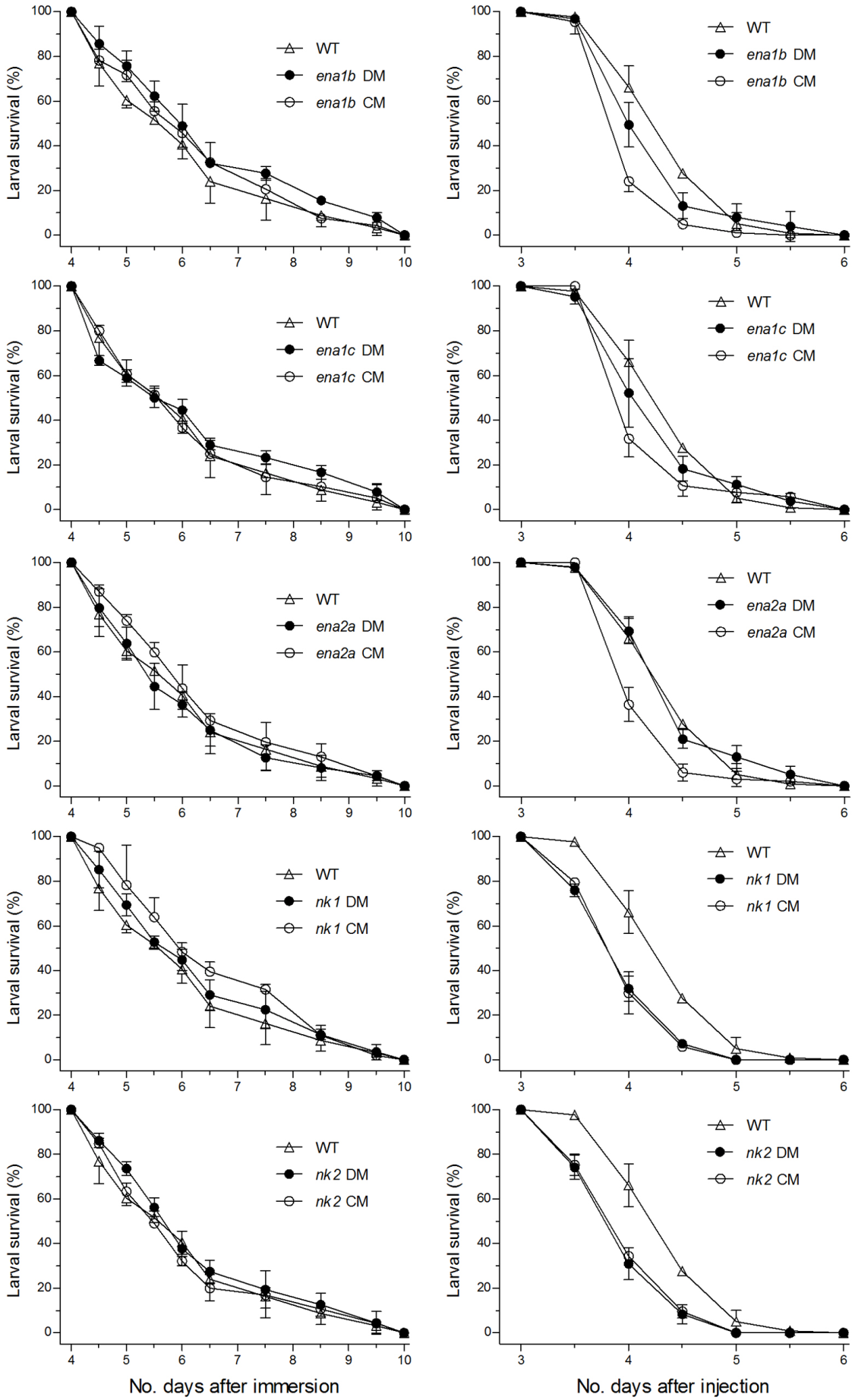


**Fig. S8.** Survival trends of *G. mellonella* larvae after topical application (immersion) of a 10^7^ conidia/ml suspension for normal cuticle infection (left panels) or intrahaemocoel injection of ~500 conidia per larva for cuticle- bypassing infection (right panels). DM, deletion mutant. CM, complemented mutant. WT, wild-type. Error bars: standard deviation of the mean from three replicates.
